# Machine learning approaches for risk prediction in aortic dissection: a systematic review and meta-analysis

**DOI:** 10.3389/fcvm.2026.1777734

**Published:** 2026-03-26

**Authors:** Yijun Mao, Qiang Liu, Hui Fan, Xiaojuan Wang

**Affiliations:** 1 Department of Nursing, Xianyang Central Hospital, Xianyang, Shaanxi, China; 2 Department of Orthopedic Surgery, Xianyang Central Hospital, Xianyang, Shaanxi, China

**Keywords:** aortic dissection, machine learning, meta-analysis, prediction model, systematic review

## Abstract

**Background:**

Aortic dissection (AD) is a life-threatening cardiovascular emergency with high morbidity and mortality. Accurate risk prediction is essential for timely intervention, yet traditional statistical models often fail to capture the complex, nonlinear interactions inherent in AD pathophysiology. In recent years, machine learning (ML) has emerged as a promising approach to improve prognostic accuracy. However, the overall performance, methodological quality, and clinical applicability of ML-based prediction models for AD have not been comprehensively evaluated.

**Objective:**

This systematic review and meta-analysis followed PRISMA, CHARMS, and TRIPOD guidelines and was registered with PROSPERO (CRD420251154262). Six major databases (PubMed, Web of Science, Cochrane Library, Embase, CNKI, Wanfang) were searched from inception to September 30, 2025. Studies developing or validating ML models for predicting adverse outcomes in AD were included. Data extraction adhered to CHARMS, and risk of bias was assessed using PROBAST. Meta-analysis synthesized C-statistics (AUC) using fixed- or random-effects models depending on heterogeneity. Subgroup, sensitivity, and publication bias analyses were performed.

**Results:**

Forty studies were included, covering outcomes such as early mortality, long-term mortality, acute kidney injury (AKI), neurological complications, gastrointestinal bleeding, mesenteric malperfusion, and composite adverse events. ML algorithms included random forest, SVM, XGBoost, LightGBM, neural networks, and ensemble approaches. The pooled C-statistic demonstrated excellent discriminative performance for early mortality (0.891, 95% CI: 0.854–0.927) and long-term mortality (0.847, 95% CI: 0.794–0.900), and strong performance for AKI prediction (0.825, 95% CI: 0.756–0.894). Many complication-specific models achieved AUCs above 0.90. However, these estimates must be interpreted with extreme caution. Significant heterogeneity was observed across analyses (*I*^2^ = 61.3–78.8%), and the PROBAST assessment revealed that 100% (40/40) of studies were at high or unclear risk of bias, predominantly due to deficiencies in the analysis domain (e.g., inadequate events-per-variable, lack of external validation). Adherence to TRIPOD reporting standards was suboptimal (average 78.7%), with critical shortcomings in reporting predictor definitions (62.5% unreported), sample size justification (82.5% unreported), and full model specifications (72.5% unreported). Methodological limitations were common, including inadequate events-per-variable ratios, a near-absence of robust external validation (only 5 of 40 studies), inconsistent outcome definitions, and incomplete reporting of model specifications. Furthermore, over a quarter (27.5%) of models omitted calibration assessment, and decision-curve analysis was rarely performed, limiting insights into clinical utility.

**Conclusion:**

ML-based prediction models demonstrate strong potential for risk stratification in AD across multiple clinically relevant outcomes. However, current evidence does not justify their routine clinical implementation. The high reported performance metrics are likely optimistic estimates derived from methodologically weak studies. Future research should emphasize rigorous analytic frameworks, standardized outcome definitions, transparent reporting, and, most critically, multicenter external validation before these tools can be considered for real-world clinical utility.

**Systematic Review Registration:**

https://www.crd.york.ac.uk/PROSPERO/view/CRD420251154262, identifier CRD420251154262.

## 1 Introduction

Aortic dissection (AD) represents one of the most lethal cardiovascular emergencies, with mortality rates exceeding 1% per hour during the initial onset if untreated (1). The condition poses significant diagnostic and therapeutic challenges due to its diverse clinical presentations, rapid progression, and complex management considerations (2). Despite advances in surgical techniques and medical management, AD continues to carry substantial morbidity and mortality, underscoring the critical need for accurate risk stratification tools to guide clinical decision-making (3).

Traditional risk prediction models in AD, predominantly based on conventional statistical approaches such as logistic regression, have demonstrated limited discriminative ability and generalizability. These limitations stem from the inherent complexity of AD pathophysiology, which involves multiple interacting clinical, anatomical, and hemodynamic factors that may exhibit non-linear relationships (4). The emergence of machine learning (ML) methodologies offers promising alternatives, with the potential to capture complex patterns in high-dimensional clinical data and improve predictive accuracy (4).

In recent years, numerous studies have explored the application of various ML algorithms—including random forests, support vector machines, neural networks, and ensemble methods—for predicting adverse outcomes in AD patients (5–7). These investigations have targeted diverse endpoints ranging from mortality and specific complications to composite outcomes. However, the literature remains fragmented, with individual studies typically focusing on single institutions or specific patient subgroups, employing heterogeneous methodologies, and reporting variable performance metrics.

The growing body of evidence necessitates a comprehensive systematic assessment to evaluate the aggregate performance of ML models in AD risk prediction. Furthermore, understanding the methodological quality and reporting completeness of these studies is essential for assessing their readiness for clinical implementation. Previous systematic reviews in cardiovascular prediction modeling have highlighted significant methodological limitations and reporting deficiencies that may undermine the validity and generalizability of published models (8, 9).

This systematic review and meta-analysis aims to address these gaps by: (1) synthesizing the discriminative performance of ML models for predicting key adverse outcomes in AD patients; (2) assessing the methodological quality and risk of bias of included studies; (3) evaluating adherence to established reporting standards; and (4) identifying sources of heterogeneity through comprehensive subgroup analyses. The findings will provide valuable insights for researchers, clinicians, and policy makers regarding the current state of ML-based risk prediction in AD and guide future research directions in this rapidly evolving field.

## 2 Methods

This systematic review and meta-analysis were conducted in accordance with the Preferred Reporting Items for Systematic Reviews and Meta-Analyses (PRISMA), the Critical Appraisal and Data Extraction for Systematic Reviews of Prediction Modelling Studies (CHARMS) checklist, and the Transparent Reporting of a Multivariable Prediction Model for Individual Prognosis or Diagnosis (TRIPOD) guidelines (see Supplementary Tables S1–S3) (10–13). The review protocol was prospectively registered with PROSPERO on September 24, 2025 and is available online (registration number: CRD420251154262). The eligibility conditions for the reviewed investigations are defined following the PICOS approach (Table 1) (14).

**Table 1:** Selection criteria of predictive modelling studies in PICOS format.

Criteria	Participants (P)	Intervention (I)	Comparison (C)	Outcomes (O)	Timeframe (T)	Settings (S)	Other limitations
Inclusion criteria	Patients with a confirmed diagnosis of aortic dissection (AD). AD Type: Any anatomical classification (Stanford Type A or B; DeBakey Type I, II, or III). Phase of Disease: Both acute and chronic/subacute phases were considered, provided the study clearly defined the cohort.	Studies that developed and/or validated (internally or externally) a risk prediction model for outcomes in aortic dissection (e.g., in-hospital mortality, composite adverse events) using machine learning (ML) algorithms*.	Not applicable (Systematic review of prediction model studies, typically without a direct comparator intervention).	Studies reporting at least one quantitative measure of model performance. Mortality: In-hospital, 30-day, or long-term (e.g., 1-year, 3-year) mortality. Specific Complications: e.g., Acute kidney injury (AKI), neurological complications (stroke, cerebral complications), gastrointestinal bleeding, mesenteric malperfusion, pulmonary complications. Composite Outcomes: e.g., Major adverse events (MAEs), a composite of mortality and aortic-related events. Performance Metrics: Primarily discrimination^†^ (e.g., AUC/C-statistic), but also accuracy, sensitivity, specificity, and calibration measures (e.g., calibration plots, Brier score, Hosmer-Lemeshow test).	From each database's inception to September 30, 2025	All clinical settings involved in the management of aortic dissection, including emergency departments, cardiology wards, intensive care units, and surgical centers, across any country income level.	Language: English and Chinese. Study Type: Full-text articles in peer-reviewed journals.
Exclusion criteria	Patients with other aortic conditions (e.g., aortic aneurysm, intramural hematoma without dissection, penetrating aortic ulcer). Patients with traumatic aortic injury. Studies focused exclusively on non-aortic cardiovascular diseases.	Studies that only used traditional statistical methods* (e.g., logistic regression) without an ML component; studies that applied existing models without developing or validating a new one.	N/A	Studies that did not report quantitative performance metrics for the prediction model.	Studies published after the search date.	No restrictions based on clinical setting.	Reviews, editorials, conference abstracts, case reports (<10 patients), animal studies, and studies in languages other than English or Chinese.

* We defined “machine learning” (ML) models as those employing algorithms capable of learning complex, non-linear relationships from data without explicit rule-based programming, including but not limited to random forests, support vector machines, gradient boosting machines (e.g., XGBoost, LightGBM), neural networks, and ensemble methods. In contrast, “traditional statistical models” were defined as regression-based approaches relying on pre-specified functional forms and assumptions about the data distribution, such as standard (unregularized) logistic regression or Cox proportional hazards models.

^†^ In this review, the terms C-statistic (concordance statistic) and AUC (area under the receiver operating characteristic curve) were treated as equivalent measures of discriminative performance. For binary outcomes (e.g., early mortality, AKI), the AUC was derived directly from ROC analysis. For time-to-event outcomes (e.g., long-term mortality), studies reported the C-index, which is analogous to the AUC for censored data and quantifies the probability that a model correctly ranks the survival times of two randomly selected patients. Both metrics were pooled together under the common label of “C-statistic” in our meta-analysis, as they share the same interpretation (range 0.5–1.0, with higher values indicating better discrimination) and are mathematically equivalent when no censoring is present. To ensure comparability, we only included C-indices derived from models that were evaluated using time-dependent ROC or Harrell's C-index with appropriate handling of censoring. Studies reporting only Harrell's C without confidence intervals or standard errors were excluded unless these could be calculated from reported sample sizes and event counts using validated methods (e.g., based on the standard error formula for the C-index).

### Participants (P)

Studies involving patients with a confirmed diagnosis of AD. All anatomical classifications (Stanford Type A or B; DeBakey Type I, II, or III) and phases of the disease (acute and chronic/subacute) were considered, provided the study clearly defined the cohort. No restrictions were placed on age, gender, ethnicity, or geographic location.

### Intervention (I)

Studies that developed or validated risk prediction models for adverse outcomes in AD using machine learning algorithms. Both internally and externally validated models were eligible. Studies using only traditional statistical methods without machine learning components were excluded.

Studies reporting at least one quantitative measure of prediction model performance, including:

- Discrimination: e.g., Area under the curve (AUC) or C-statistic.
- Calibration: e.g., Calibration plots, Brier score, or Hosmer–Lemeshow test.
- Clinical outcomes: Such as mortality (in-hospital, 30-day, or long-term), specific complications (e.g., acute kidney injury, stroke, gastrointestinal bleeding, mesenteric malperfusion, pulmonary complications), or composite outcomes (e.g., major adverse events).
- Clinical utility: e.g., Decision curve analysis.

### Outcomes (O)

Performance metrics of prediction models, including discrimination (e.g., area under the curve), calibration (e.g., calibration plots or statistics), and clinical utility (e.g., decision curve analysis).

### Timeframe (T)

The database search encompassed records from each database's inception through September 30, 2025.

### Settings (S)

All clinical settings, including emergency departments, cardiology wards, vascular surgery units, and intensive care units, across high-income, middle-income, and low-income countries were included. Studies published in English or Chinese were eligible for inclusion.

### 2.1 Search strategy

A comprehensive literature search was conducted using PubMed, Web of Science, Cochrane Library, Embase, CNKI, and Wanfang databases, covering publications from database inception through September 30, 2025. Details of the search strategy, including keywords, and the inclusion and exclusion criteria, are provided in the Supplementary Table S4. It is important to note that the inclusion of Master's theses and dissertations, may introduce variability in study quality. While these sources can provide valuable data and reduce publication bias by including grey literature, they may also be associated with lower methodological rigor compared to peer-reviewed publications.

### 2.2 Data extraction and quality assessment

Data extraction was performed independently by two reviewers using a standardized form based on the CHARMS checklist and included the following domains:

- Participants: Demographic and clinical characteristics, including the proportion of patients with conditions such as in-hospital mortality.
- Study design: Key design features (e.g., prospective or retrospective cohort design, sample size).
- Outcome measures: Definitions of the predicted outcomes in the risk prediction models.
- Model development: Methods for predictor selection and model validation.
- Model performance: Discrimination measures, such as the c-statistic.

The risk of bias (ROB) and applicability of each included study were assessed using the Prediction model Risk Of Bias ASsessment Tool (PROBAST) (15). This tool evaluates four key domains: participants, predictors, outcome, and analysis. Within each domain, signaling questions were used to rate the ROB as “low,” “high,” or “unclear.” The overall ROB for each study was determined based on the domain-level ratings. The assessment was conducted independently by two reviewers, and any disagreements were resolved through consensus or consultation with a third reviewer.

### 2.3 Data synthesis and statistic analysis

A meta-analysis was conducted to assess the discriminative performance of prognostic models for aortic dissection, as measured by the area under the receiver operating characteristic curve (AUC). To be eligible for inclusion, studies were required to report or allow calculation of the C-statistic and corresponding 95% confidence intervals (CIs) for models. Where confidence intervals were not reported in the original studies, we calculated them using the standard error of the AUC or, if unavailable, derived them from reported sample sizes and event rates using established methods (e.g., Hanley & McNeil). Where multiple models were developed within the same study, the model evaluated using internal validation was selected for inclusion in the meta-analysis, with preference given to the model reporting the C-statistic based on the validation dataset rather than the training dataset. This approach was adopted to minimize overfitting and provide a more realistic estimate of model performance. Heterogeneity was assessed using the Cochran's *Q* test and quantified with the *I*^2^ statistic. Substantial heterogeneity was predefined as an *I*^2^ > 50% and a *P*-value for the *Q* test <0.10. Given the anticipated clinical and methodological diversity across studies (e.g., differences in populations, outcome definitions, and ML algorithms), we planned to use a random-effects model (DerSimonian and Laird method) for all primary analyses *a priori*, regardless of the level of statistical heterogeneity, to provide a more conservative and generalizable pooled estimate. Given the anticipated heterogeneity across studies in terms of populations, outcomes, and ML algorithms, we used random-effects models for all primary analyses. The pooled C-statistics from these models should be interpreted as the average effect across a distribution of true effects, rather than a single common effect. To better characterize the uncertainty and range of this distribution, we calculated 95% prediction intervals for the pooled estimates, which estimate the range in which the true C-statistic of a future study would lie. To explore potential sources of heterogeneity, we conducted pre-specified subgroup analyses based on clinically and methodologically relevant variables. These included: (1) patient population (e.g., AAD vs. ATAAD), (2) sample size (using a median split or predefined cutoffs like <500 vs. ≥500), (3) events-per-variable (EPV) ratio (<10, 10–20, >20), and (4) validation strategy (e.g., internal validation only vs. any external validation). These analyses were only performed for outcome categories with a sufficient number of studies (typically ≥5). Funnel plot asymmetry was assessed both visually and statistically using Egger's linear regression test, with *P* < 0.05 considered indicative of potential publication bias. All analyses were performed in Review Manager (version 5.4; The Cochrane Collaboration, London, UK) and R (version 4.4.1; R Foundation for Statistical Computing, Vienna, Austria) using the meta and metafor packages.

## 3 Results

### 3.1 Search results

**Figure 1:**
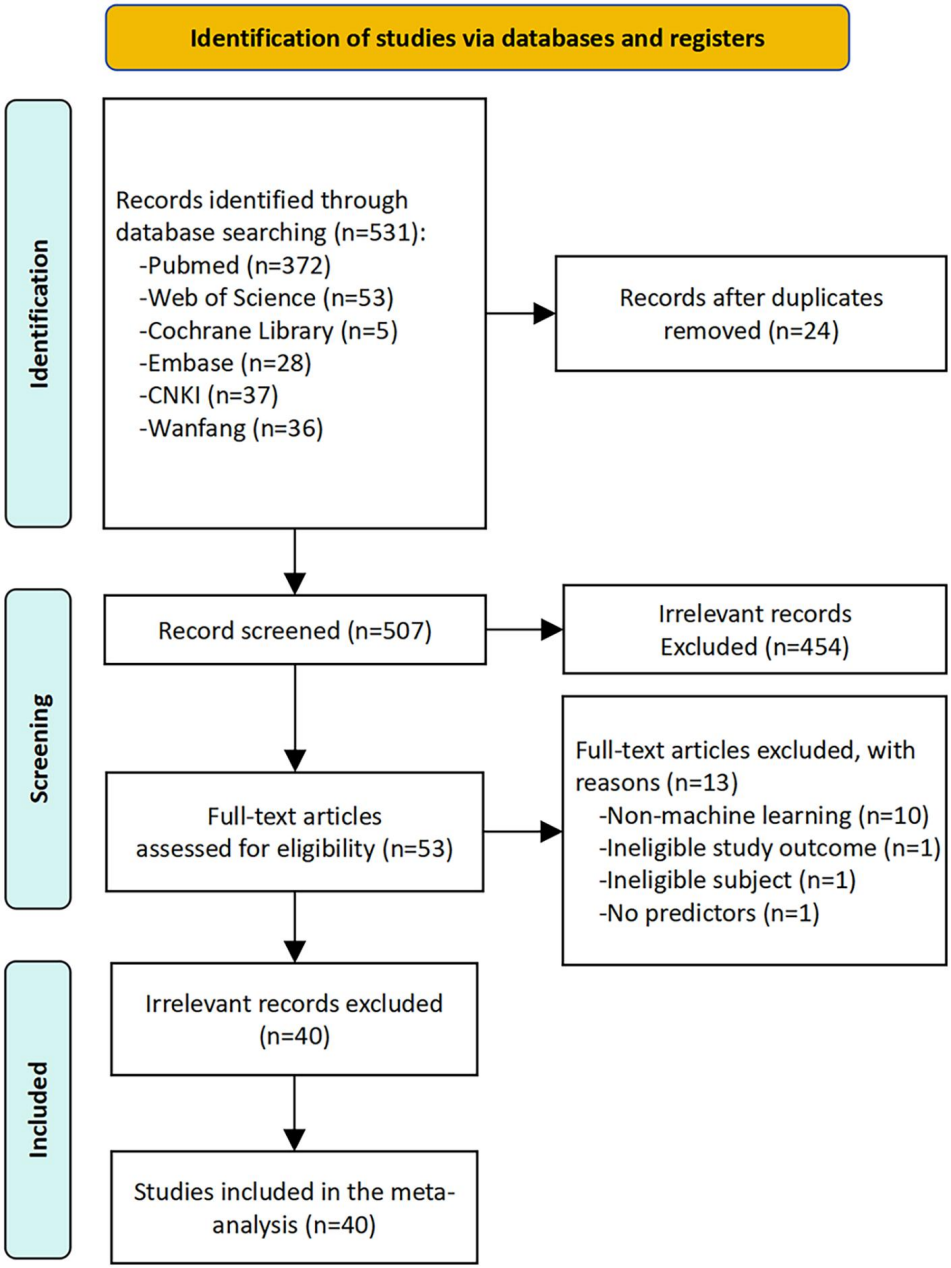
The preferred reporting items for systematic review and meta-analysis (PRISMA) flowchart of studies.

Following the removal of duplicates, the literature search yielded 507 unique records. Of these, 40 studies met the inclusion criteria and were included in the systematic review and meta-analysis (16–55) (Figure 1). Detailed reasons for the exclusion of full-text articles are provided in Supplementary File 1.

### 3.2 Included studies

This review included 40 studies published between 2021 and 2025. The literature was dominated by retrospective designs (*n* = 38), supplemented by one prospective study and one ambidirectional cohort study (which incorporates both retrospective and prospective components). Sample sizes ranged from 119 to 5,449 patients. The validation approaches varied: 31 studies reported internal validation, while 7 involved external validation in independent cohorts. Additionally, 2 studies were dedicated solely to model development without subsequent validation. A detailed summary of study characteristics is provided in Table 2 and Supplementary Table S5. Of the 40 included studies, 4 (10.0%) were identified from Chinese-language databases (CNKI and Wanfang) and comprised Master's theses or dissertations that had not undergone formal peer review. The inclusion of non-peer-reviewed grey literature may have implications for study quality; however, it also broadens the evidence base and reduces the risk of publication bias by incorporating studies that might otherwise remain unpublished due to negative or null findings.

**Table 2:** Basic characteristics of the included studies.

First author, year	Type	Source data	Region	Patient recruitment years	Participants	Main outcome	Events/sample size (%)	EPV
Cai, 2025	D/V	RC	China	2017–2020	TAAD	Mortality at follow-up	53/171 (31.0%)	8
Chen, 2023	D/V	RC	China	2017–2021	ATAAD	Cerebral complications	27/145 (18.6%)	4
Chen, 2021	D/V	RC	China	2016–2019	ATAAD	LOS	-/353	NI
Chen, 2025	D/V	RC	China	2015–2022	ATAAD	Mortality at 30 d	84/925 (9.1%)	11
Chen, 2025	D/V	RC	China	2018–2023	ATAAD	AKI	586/1350 (43.4%)	59
Dai, 2023	D/V	RC	China	2019–2022	ATAAD	AKI	191/265 (72.1%)	21
Dong, 2021	D/V	RC	China	2011–2018	TBAD	Reintervention	68/192 (35.4%)	10
Guo, 2021	D/V	RC	China	2015–2018	AAD	In-hospital mortality	273/1344 (20.3%)	14
Guo, 2022	D/V	PC	China	2019	AAD	Mortality at 1 y	142/695 (20.4%)	16
He, 2025	D/V	AC	China	2015–2024	AAD	Mortality and aortic-related composite endpoint at follow-up	44/405 (26.7%)	4
Jiang, 2023	D/V	RC	China	2013–2021	ATAAD	Mortality at 30 d	205/1411 (14.5%)	23
Jin, 2025	D/V	RC	China	2016–2022	AAD	MMP	87/5449 (1.6%)	17
Jin, 2025	D/V	RC	China	2015–2022	AAD	MMP	105/525	26
Lei, 2024	D/V	RC	the US	2008–2019	AD	In-hospital mortality	152/1144 (13.3%)	6
Li, 2025	D/V	RC	China	2020–2024	ATAAD	Postoperative CRRT	64/588 (10.9%)	11
Li, 2025	D/V	RC	China	2019–2024	TAAD	postoperative GIB	63/525 (12.0%)	7
Li, 2025	D/V	RC	China	2020–2024	AD	prolonged LOS (exceeding 30 days)	111/506 (21.5%)	22
Li, 2024	D	RC	China	2015–2022	ATAAD	Mortality at 30 d	54/329 (16.5%)	6
Li, 2022	D/V	RC	China	2019–2021	AAD	AKI	120/173 (69.4%)* 81/283 (28.6%)**	20
Lin, 2023	D/V	RC	China	2012–2017	ATAAD	death due to the dissection rupturing within 72 h after the CTA	100/200	10
Liu, 2024	D/V	RC	China	2018–2021	ATAAD	AKI	131/572 (22.9%)	16
Lu, 2024	D/V	RC	China	2015–2022	uTBAD	Postoperative adverse outcomes	182/369 (49.3%)	15
Luo, 2025	D/V	RC	China	2018–2023	ATAAD	Major adverse outcomes	160/635 (25.2%)	15
Ma, 2024	D	RC	China	2016–2021	ATAAD	AGI	60/188 (31.9%)	15
Pang, 2024	D/V	RC	China	2016–2021	TBAD	Complicated TBAD	44/180 (24.5%)	9
Pei, 2021	D/V	RC	China	2013–2017	TAAD	MAEs	426/1641 (26.0%)	61
Song, 2024	D/V	RC	China	2019–2023	ATAAD	In-hospital mortality	104/688 (15.1%)	21
Sun, 2024	D/V	RC	China	2014–2023	ATAAD	Mortality at 3 y	-/976	NI
Wang, 2023	D/V	RC	China	2015–2017	ATAAD	MS-ARDS within 24 h	243/594 (40.9%)	22
Wang, 2023	D/V	RC	China	2008–2020	TBAD	Adverse aortic events	26/119 (21.8%)	4
Wei, 2025	D/V	RC	China	2001–2019	AAD	In-hospital AKI	84/325 (25.9%)	9
Wei, 2025	D/V	RC	China	2017–2024	ATAAD	postoperative pulmonary complications	96/340 (28.2%)	9
Wen, 2025	D/V	RC	China	2020–2023	AAD	Postoperative reintubation	107/861 (12.4%)	27
Wu, 2023	D/V	RC	China	2004–2018	AAD	In-hospital mortality	55/380 (14.5%)	11
Xie, 2024	D/V	RC	China	2018–2022	AAD	Postoperative adverse outcomes	22/380 (5.8%)^†^ 167/380 (44.0%)^‡^	19
Xie, 2024	D/V	RC	China	2018–2022	AAD with malnutrition	MAEs	40/308 (13.0%)^†^ 87/308 (28.3%)^‡^	12
Zhang, 2024	D/V	RC	China	2017–2022	TAAD	Mortality at 1 y	106/289 (36.7%)	13
Zhang, 2025	D/V	RC	China	2015–2020	ATAAD	Mortality at 30 d	37/640 (5.8%)	4
Zhang, 2025	D/V	RC	China	2013–2023	AAD	Mortality at 30 d	145/782 (18.5%)	10
Zhao, 2021	D/V	RC	China	2015–2019	ATAAD	Pre-operation AIS	86/300 (28.7%)	22

AAD, acute aortic dissection; AC, ambidirectional cohort study; AD, aortic dissection; AGI, acute gastrointestinal injury; AIS, acute ischemic stroke; AKI, acute kidney injury; ATAAD, acute type A aortic dissection; CRRT, continuous renal replacement therapy; D, development study; GIB, Gastrointestinal bleeding; MAEs, major adverse events; MMP, mesenteric malperfusion; MS-ARDS, moderate to severe acute respiratory distress syndrome; PC, prospective cohort study; RC, retrospective cohort study; TAAD, type A aortic dissection; TBAD, type B aortic dissection; uTBAD, uncomplicated Stanford type B aortic dissection; V, validation study.

* TAAAD

** TBAAD

^†^ In-hospital mortality

^‡^ Postoperative adverse outcome rate.

Formula: EPV = Number of Events/Number of Predictor Variables.

According to the PROBAST guideline, studies with an EPV <10 were considered to be at high risk of overfitting.

### 3.3 Study characteristics

The included studies developed ML models to predict a diverse range of clinically significant outcomes following aortic dissection. A total of 40 distinct prediction models were identified across the 40 included studies. The most common outcome category was postoperative organ injury and specific complications (15 models, 37.5%), with acute kidney injury being the most frequently predicted single complication (5 models). This category also encompassed cerebral complications (e.g., stroke), pulmonary complications, mesenteric malperfusion, acute gastrointestinal injury, gastrointestinal bleeding, and the need for continuous renal replacement therapy. The second most frequent outcome was mortality (14 models, 35.0%), which was further stratified into early mortality (in-hospital or 30-day; 9 models) and longer-term mortality (1–3 years; 5 models). Composite outcomes, primarily combining mortality with other major adverse events, were used in 11 models (27.5%). The remaining models predicted other endpoints such as prolonged hospital stay, aortic reintervention, and progression to complicated type B aortic dissection. A detailed breakdown of predicted outcomes is provided in Table 2.

The included studies utilized a diverse array of machine learning algorithms for model development. Among the specified models, the most frequently employed were Random Forest (RF) (60.0%), Support Vector Machine (SVM) (60.0%), and XGBoost (applied in 52.2% of studies). Logistic Regression (LR) was also a commonly used baseline model (70.0%), while various neural network architectures, including K-Nearest Neighbors (KNN) and Multi-Layer Perceptrons (MLP) were employed in 32.5% and 15.0% of the studies, respectively. Data preprocessing and validation methodologies varied across the studies. For handling missing data, a common strategy was to exclude variables with a missingness proportion exceeding a predefined threshold (ranging from 10% to 30%), followed by multiple imputation for the remaining variables, which was employed in 12 studies (30.0%). The LASSO (Least Absolute Shrinkage and Selection Operator) regression was the predominant technique for variable selection, featuring in 24 studies (60.0%).

The evaluation metrics used to assess model performance varied across the included studies. Among the 40 studies reviewed, the AUC was universally reported (100%). In contrast, other common classification metrics were reported less frequently: accuracy was provided in 22 studies (55.0%), sensitivity and specificity in 17 (42.5%), and precision in 11 (27.5%). The Brier score was reported in 13 studies (32.5%), while net reclassification improvement (NRI) and integrated discrimination improvement (IDI) were each reported in only 2 studies (5.0%). A variety of model validation techniques were employed across the studies. The most frequently used method was cross-validation (50.0%), with 10-fold cross-validation being the most common variant, followed by 5-fold cross-validation. Hold-out validation was used in 16 studies (42.5%), and external temporal validation was applied in 5 studies (12.5%). Bootstrapping was used in 4 studies (10.0%). Some studies employed more than one validation approach to enhance the robustness of their performance estimates. Calibration of the predictive models was assessed in 29 studies (72.5%). The most common calibration metrics were calibration curves (19 studies, 47.5%) and the Brier score (15 studies, 37.5%). The Hosmer–Lemeshow test was used in 7 studies (17.5%). A minority of studies reported both discrimination and calibration metrics, facilitating a more comprehensive evaluation of model performance. A detailed overview of modeling methodologies for each individual study is provided in Table 3.

**Table 3:** Domains of performance of aortic dissection risk prediction models.

Author (year)	Missing data handling	Continuous variable processing method	Variable selection	Model development method	Calibration method	Validation method	Model performance	Model presentation
Cai, 2025	Random forest algorithms was applied to variables with <20% missing data, whereas those with a proportion exceeding 20% were excluded.	Continuity	LASSO Cox regression analysis, univariate analysis and correlation analysis	SVM	Brier score	Hold-out validation, 10-fold cross-validation and external temporal validation	A: 0.914 (0.981, 0.920) B1: 0.853 (0.850, 0.862) B2: 0.877 (0.870, 0.898)	Risk score
Chen, 2023	Excluded	Continuity	LASSO	LR, KNN, RF, GBM, SVM, MLP	Brier score and Hosmer-Lemeshow test	Hold-out validation	B: 0.828 (0.585, 0.902)	NI
Chen, 2021	Features with missing values more than 20% were excluded. Missing data were assumed to be missing at random and were imputed using 10-fold multiple imputation by chained equations.	Continuity	Kendall correlation coefficient	NB, LR, DT, RF, GBDT	NI	5-fold cross-validation	A: 0.991 (0.978, 1.000) B: 0.837 (0.766, 0.908)	NI
Chen, 2025	MissForest imputation method was applied to variables with <10% missing data, whereas those with a proportion exceeding 10% were excluded.	Continuity	Univariate and multivariate binary logistic regression analyses, DT, RF, XGBoost, SVM	LR, DT, RF, XGBoost, SVM	Brier score, calibration curve and Hosmer-Lemeshow test	Hold-out validation	A: 0.842 (0.780, 0.910) B: 0.782 (0.698, 0.860)	Web-based calculator
Chen, 2025	Variables with more than 20% missing data were excluded from further analysis. For variables with missing data below this threshold, multiple imputation was employed to minimize bias.	Continuity	NI	GBM, LightGBM, RF, KNN, MLP-NN, NB, LR	Brier score, calibration curve	10-fold cross-validation	B: 0.874 (0.831, 0.918)	Web-based calculator
Dai, 2023	K-nearest neighbor (KNN) method was applied to fill in the missing data.	Continuity	LR, LASSO	LR with L2, RFC, SVM, XGBoost	Brier score	10-fold cross-validation	B: 0.800 (0.683, 0.917)	NI
Dong, 2021	Excluded	Continuity	LASSO	XGBoost, AdaBoost, NB, LR, RF, KNN, K-SVM, BPNN	Calibration curve	5-fold cross-validation and bootstrapping	A: 0.848 (0.720, 0.975) B: 0.802 (0.689, 0.915)	Nomogram
Guo, 2021	Imputed values (which were combined using Rubin's rules) were used to impute variables with <10% missing data, whereas those with a proportion exceeding 10% were excluded.	Continuity	NI	LR, DT, k-NN, GNB, XGBoost	NI	10-fold cross-validation	B: 0.927 (0.860, 0.968)	NI
Guo, 2022	Multiple imputation was applied to variables with <20% missing data, whereas those with a proportion exceeding 20% were excluded.	Continuity	LASSO	COX, NN	Brier score	10-fold cross-validation	A: 0.912 (0.866, 0.957) B: 0.899 (0.824, 0.975)	Nomogram
He, 2025	Multiple imputation was applied to variables with <10% missing data, whereas those with a proportion exceeding 10% were excluded.	Continuity	CoxBoost, StepCox, RSF, LASSO	LASSO, Ridge, ElasticNet, StepCox, RSF, SuperPC, plsReox, GBM, CoxBoost, survival-SVM	Calibration curve	Hold-out validation	B1a: 0.826 (0.760, 0.891) B1b: 0.815 (0.758, 0.871)	Web-based calculator
Jiang, 2023	Multiple imputation was applied to variables with <10% missing data, whereas those with a proportion exceeding 20% were excluded.	Continuity	Backward stepwise regression, LASSO, Best subset regression, RF, AdaBoost, Weighted k-NN, SVM, NN	LR, LASSO, BSS, RF, AdaBoost, Weighted k-NN, SVM, NN	NI	5-fold cross-validation	B: 0.841 (0.771, 0.911)	Nomogram
Jin, 2025	We addressed missing values through multiple imputation	Continuity	LASSO	LR, SVC, RF, XGBoost, NB, MLP	Calibration curve and Brier score	5-fold cross-validation	A: 0.888 (0.887, 0.889) B: 0.797 (0.794, 0.800)	NI
Jin, 2025	Variables with >10% missing data were excluded before further analysis. We subsequently addressed missing values in clinical variables identified during the model-building process using multiple imputation	Continuity	Univariate and multivariate analyses	LR, deep learning	Brier score	5-fold cross-validation and external temporal validation	B2: 0.780 (0.777, 0.785)	NI
Lei, 2024	Excluded	Continuity	Univariate analyses	LR, DT, RF, XGBoost	NI	Hold-out validation and external temporal validation	B1: 0.870 (0.744, 0.996) B2: 0.767 (0.658, 0.876)	Web-based calculator
Li, 2025	Variables with missing data exceeding 30% were excluded. For variables with less than 30% missing data, missing values were imputed using appropriate methods: the mean was used for continuous variables, and the mode was used for categorical variables.	Continuity	LASSO	XGBoost, LR, DT, GNB, MLP, KNN, SVM	Calibration curve	5-fold cross-validation	B: 0.96 (0.93, 0.99)	SHAP force plot
Li, 2025	Variables with missing data exceeding 30% were excluded. For the remaining missing variables, imputation was performed using the MissForest package in R 4.3.3 to ensure the completeness and accuracy of the dataset.	Continuity	LASSO	RF, KNN, SVM, DT	Calibration curve	5-fold cross-validation	B: 0.933 (0.840, 0.997)	SHAP dependency plot
Li, 2025	Multiple imputation was applied to variables with <30% missing data, whereas those with a proportion exceeding 30% were excluded.	Continuity	Boruta algorithm	XGBoost, AdaBoost, KNN, LR, LightGBM, GNB, MLP, CNB, SVM	Calibration curve	Hold-out validation	A: 0.960 (0.940, 0.970) B: 0.710 (0.620, 0.800)	SHAP
Li, 2024	NI	Continuity	LASSO and PCA	LR, SVM, RF, GBM, SL	Brier score	NI	A: 0.959 (0.927, 0.992)	NI
Li, 2022	NI	Continuity	Recursive Feature Elimination (RFE) and 5-fold cross-validation	DT, RF, XGBoost, LightGBM, LR	Calibration curve and Brier score	Bootstrap	A1: 0.760 (0.630, 0.881) A2: 0.734 (0.623, 0.847)	NI
Lin, 2023	NI	Made binary using cutoff points	forward step method	LR, CNN, RF, SVM	Calibration curve and Hosmer-Lemeshow test	Bootstrap	B: 0.990 (0.950, 0.990)	NI
Liu, 2024	NI	Continuity	LASSO, SVM-RFE, RF	ANN, LR	Calibration curve	Bootstrap and 10-fold cross-validation	B: 0.916	NI
Lu, 2024	NI	Continuity	ANOVA and LASSO	XGBoost	Calibration curve	Hold-out validation and external temporal validation	A: 1.000 (1.000, 1.000) B1: 0.990 (0.966, 1.000) B2: 0.985 (0.965, 1.000)	NI
Luo, 2025	NI	Continuity	elastic network, Lasso, Ridge, stepwise Cox, CoxBoost, RSF, SVM, partial least squares regression for Cox, GBM, and supervised principal components	elastic network, Lasso, Ridge, stepwise Cox, CoxBoost, RSF, SVM, partial least squares regression for Cox, GBM, and supervised principal components	Calibration curve and Brier score	Hold-out validation	A: 0.957 B: 0.882	Web-based calculator
Ma, 2024	NI	Continuity	LR	RF	NI	NI	A: 0.990 (0.966, 1.000)	NI
Pang, 2024	NI	Continuity	LASSO, 10-fold cross-validation and multivariable LR	LR, SVM, KNN, RF, DT, XGBoost	NI	Hold-out validation	A: 0.877 (0.803, 0.952) B: 0.818 (0.641, 0.995)	NI
Pei, 2021	Multiple imputation was applied to handle missing data.	Continuity	stepwise forward selection, LASSO and XGBoost	LR	Hosmer-Lemeshow test	Hold-out validation	B: 0.766 (0.718, 0.734)	Nomogram
Song, 2024	Multiple imputation was applied to variables with <20% missing data, whereas those with a proportion exceeding 20% were excluded.	Continuity	RF	LR	Hosmer-Lemeshow test and calibration curve	Hold-out validation	A: 0.759 (0.735, 0.851) B: 0.856 (0.791, 0.922)	Nomogram
Sun, 2024	Multiple imputation was applied to variables with <10% missing data, whereas those with a proportion exceeding 10% were excluded.	Continuity	LASSO	RSF, COX, SVM, XGBoost, Deepsurv	Brier score	Hold-out validation	A: 0.930 B: 0.756	Nomogram
Wang, 2023	Variables missing more than 10% of the values were excluded; for variables missing less than 10% of the values, the data were interpolated (5 times interpolation).	Continuity	univariate logistic analysis	XGBoost, LR	NI	Hold-out validation	B: 0.797	Nomogram
Wang, 2023	Excluded	Continuity	LASSO	LR, NB, DT, CART, Bootstrap	Calibration curve and Hosmer-Lemeshow test	10-fold cross-validation	A: 0.942 B: 0.852	Nomogram
Wei, 2025	The data were preprocessed by removing features with more than 20% missing values, and the remaining missing values were added to the dataset using the predictive mean matching method (PMM) for multiple imputations.	Continuity	univariate and multivariate logistic regression analyses	SVM, GBM, NNET, XGBoost, KNN, LightGBM, CatBoost	NI	10-fold cross-validation	A: 0.876 (0.833, 0.997) B1: 0.723 (0.610, 0.837) B2: 0.712 (0.604, 0.791)	NI
Wei, 2025	The data were preprocessed by removing features with more than 10% missing values, and the remaining missing values were added to the dataset using the predictive mean matching method (PMM) for multiple imputations.	Continuity	univariate and multivariate Logistic Regression (LR)	LR, SVM, GBM, NNET, XGBoost, KNN, AdaBoost, LightGBM, CatBoost	Hosmer-Lemeshow test	10-fold cross-validation	A: 0.888 (0.849, 0.928) B: 0.763 (0.667, 0.859)	NI
Wen, 2025	For continuous variables, missing values were imputed using the mean if the data were normally distributed or the median if the data were skewed. For categorical variables, missing values were replaced with the most frequent category. If the proportion of missing values for a variable exceeded 30% of the total observations, the variable was excluded.	Continuity	LASSO	MLR, DT, RF, XGBoost, SVM, KNN, LightGBM	Calibration plots	10-fold cross-validation	A: 0.984 B1: 0.969 B2: 0.990	Web-based calculator
Wu, 2023	NI	Continuity	NI	LR, DT, RF, XGBoost, SVM	Calibration curve	Hold-out validation	A: 0.926 (0.855, 0.997)	Nomogram
Xie, 2024	NI	Continuity	LASSO	XGBoost, LR, RF, GNB, SVM, KNN	Calibration curve and Brier score	10-fold cross-validation	A: 0.997 (0.993, 1.000) B: 0.716 (0.668, 0.854)	NI
Xie, 2024	NI	Continuity	LASSO	XGBoost, LR, RF, MLP, SVM, KNN	Calibration curve and Brier score	10-fold cross-validation	B: 0.975 (0.940, 1.000)	NI
Zhang, 2024	NI	Continuity	LASSO, 10-fold cross-validation	LR, Treebag, GBM, AdaBoost	Calibration curve, Brier score	5-fold cross-validation	B: 0.910 (0.841, 0.962)	Web-based calculator
Zhang, 2025	ELM was employed to replace the missing data.	Continuity	Pearson correlation coefficients, XGBoost	XGBoost, LR, SVM, RF	NI	Hold-out validation	B: 0.869	NI
Zhang, 2025	Multiple imputation was applied to variables with ≤25% missing data, whereas those with a proportion exceeding 25% were excluded.	Continuity	LASSO	XGBoost	NI	Hold-out validation and external temporal validation	A: 0.928 (0.901, 0.956) B1: 0.919 (0.891, 0.949) B2: 0.941 (0.915, 0.967)	Web-based calculator
Zhao, 2021	NI	Continuity	univariate and multivariate stepwise logistic regression analysis, the 10-fold CV LASSO	SVM, RF, NN, DNN	NI	Hold-out validation	A: 0.982 (0.967, 0.997) B: 0.964 (0.932, 0.997)	NI

ANOVA, analysis of variance; ANN, artificial neural network; BSS, best subset selection; BPNN, back propagation neural networks; CART, classification and regression trees; CNN, convolutional neural network; CNB, categorical naive bayes; DT, decision tree; DNN, deep neural network; ELM, extreme learning machine; GBDT, gradient boosting decision tree; GBM, gradient boosting machine; GNB, Gaussian naive Bayes; KNN, K-nearest neighbors; K-SVM, kernel support vector machine; LASSO, least absolute shrinkage and selection operator; LR, logistic regression; LightGBM, light gradient boosting machine; MLP, multilayer perceptron; MLR, multiple logistic regression; NB, naive bayes; NN, neural network; NNET, neural network; PCA, principal component analysis; PMM, predictive mean matching; RF, random forest; RFE, recursive feature elimination; RSF, random survival forest; Ridge, ridge regression; SL, super learner; SVM, support vector machine; SVC, support vector classifier; SuperPC, supervised principal components; XGBoost, extreme gradient boosting.

**Figure 2:**
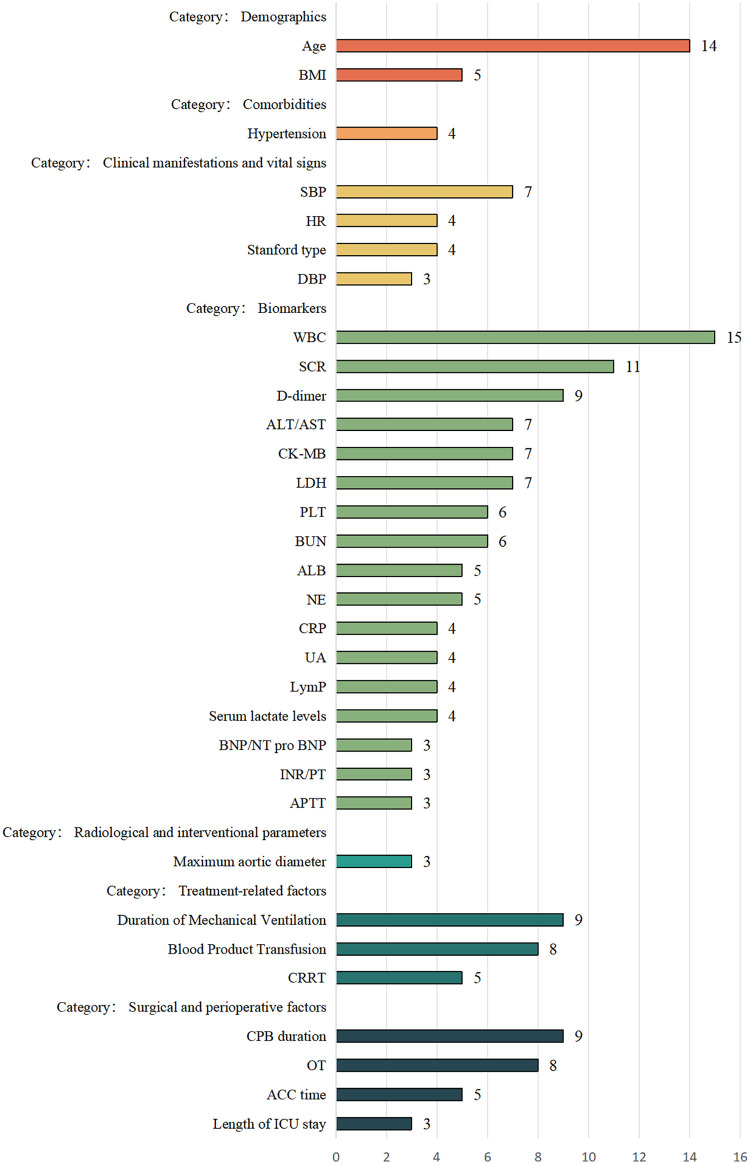
Main predictors of the included models. BMI, body mass index; SBP, systolic blood pressure; HR, heart rate; DBP, diastolic blood pressure; WBC, white blood cell count; SCR, serum creatinine; ALT/AST, alanine aminotransferase/aspartate aminotransferase; CK-MB, creatine kinase-MB; LDH, lactate dehydrogenase; PLT, platelet count; BUN, blood urea nitrogen; ALB, albumin; NE, neutrophils; CRP, C-reactive protein; UA, uric acid; LymP, lymphocyte percentage; BNP/NT pro BNP, B-type natriuretic peptide/N-terminal pro-B-type natriuretic peptide; INR, international normalized ratio; PT, prothrombin Time; APTT, activated partial thromboplastin time; CRRT, continuous renal replacement therapy; CPB, cardiopulmonary bypass; OT, operation time; ACC, aortic cross-clamp; ICU, intensive care unit.

A comprehensive set of predictors was employed across the included studies for model development. In total, 157 unique variables were utilized, which were organized into 7 distinct feature sets. Among the demographic and clinical variables, Age was the most predominant predictor, featuring in 35.0% of all models. It was followed by White Blood Cell Count (WBC) and Serum Creatinine (SCR), which were incorporated in 35.0% and 30.0% of the models, respectively. Body Mass Index (BMI) and Gender were also commonly used, appearing in 17.5% and 10.0% of the models. In contrast, lifestyle factors such as Smoking was included in only a small minority of models (10.0% each). Regarding specific comorbidities, Hypertension was the most frequently represented, present in 15.0% of the models. Other conditions like Diabetes Mellitus (DM), Heart Failure, Obstructive Lung Disease, and Congenital Disorder were each featured in 10.0% of the models. Among the key surgical and anatomical predictors, Stanford Type classification was a critical feature, included in 20.0% of the models. The Maximum Aortic Diameter was also significant, appearing in 17.5% of the models. Additionally, Operational Time (OT) and extended Cardiopulmonary Bypass (CPB) duration were prominent perioperative factors, both of which were incorporated in 17.5% of the models. A subset of models incorporated established clinical scores, with the APACHE II score being integrated into 15.0% of the models. The overview of all predictors variables are detailed in Figure 2 and Supplementary Table S6.

### 3.4 Model performance

The C-statistic (AUC-ROC) was the primary measure for assessing model performance, documented in all included studies. The sensitivity and specificity were also widely utilized, featured in 60% of the studies. NRI and IDI were less prevalent, reported in only 20% of the analyses. For evaluating calibration, the Hosmer–Lemeshow goodness-of-fit test was the most commonly applied technique (80%). A summary of the performance assessment criteria for each investigation is provided in Tables 3, 4.

**Table 4:** Comparison of model performance using discrimination and reclassification metrics.

Study	Outcome	Best model	Validation	AUC (95%CI)	Calibration metrics	Reclassification metrics	Additional performance metrics
Early mortality							
Chen, 2025	30-day mortality	SVM	Hold-out	0.782 (0.698, 0.860)	Brie*r* = 0.058; Good calibration curve fitting; HL *P* = 0.949	NRI = 0.143; IDI = 0.062	NI
Guo, 2021	In-hospital mortality	XGBoost	10-fold CV	0.927 (0.860, 0.968)	NI	NI	Acc: 91.8%; Prec: 68.3%
Jiang, 2023	30-day mortality	BSS	5-fold CV	0.841 (0.771, 0.911)	NI	NI	Acc: 86.6%; Prec: 76.9%
Lei, 2024	In-hospital mortality	RF	Hold-out + External	0.870 (0.744, 0.996)	NI	NI	NI
Li, 2024	30-day mortality	SL	NI	0.959 (0.927, 0.992)	Brie*r* = 0.046	NI	Sens: 98.9%, Spec: 75.9%
Song, 2024	In-hospital mortality	LR	Hold-out	0.856 (0.791, 0.922)	Good calibration curve fitting; HL *P* = 0.804	NI	NI
Wu, 2023	In-hospital mortality	XGBoost	Hold-out	0.926 (0.855, 0.997)	Good calibration curve fitting	NI	NI
Zhang, 2025	30-day mortality	PSO-ELM-FLXGBoost	Hold-out	0.869	NI	NI	NI
Zhang, 2025	30-day mortality	XGBoost	Hold-out + External	0.941 (0.915, 0.967)	NI	NI	Acc: 90.1%, Sens: 81.4%, Spec: 92.1%, Prec: 70.6%
Long-term mortality							
Cai, 2025	Mortality at follow-up	SVM	Hold-out + 10-fold CV + External	0.877 (0.870, 0.898)	Brie*r* = 0.132	NI	Acc: 87.7%, Prec: 82.4%
Guo, 2022	1-year mortality	NN	10-fold CV	0.899 (0.824, 0.975)	Brie*r* = 0.120	NI	Acc: 87.1%, Sens: 65.0%, Spec: 92.3%
He, 2025	Mortality and aortic-related composite endpoint at follow-up	StepCox[backward] + RSF	Hold-out	0.826 (0.760, 0.891)	Good calibration curve fitting	NI	NI
Sun, 2024	3-year mortality	RSF	Hold-out	0.756	Brie*r* = 0.137	NI	NI
Zhang, 2024	1-year mortality	Treebag	5-fold CV	0.910 (0.841, 0.962)	Brie*r* = 0.128	NI	Sens: 81.6%
Acute kidney injury							
Chen, 2025	AKI	LightGBM	10-fold CV	0.874 (0.831, 0.918)	Brier; Good calibration curve fitting	NI	NI
Dai, 2023	AKI	XGBoost	10-fold CV	0.800 (0.683, 0.917)	Brier	NI	Sens: 71.7%
Li, 2022	AKI	RF, LightGBM	Bootstrap	0.760 (0.630, 0.881) 0.734 (0.623, 0.847)	Brie*r* = 0.160 and 0.150; Good calibration curve fitting	NI	NI
Liu, 2024	AKI	ANN	10-fold CV + Bootstrap	0.916	Good calibration curve fitting	NI	Sens: 94.0%, Spec: 81.0%
Wei, 2025	AKI	CatBoost	10-fold CV	0.712 (0.604, 0.791)	NI	NI	Acc: 72.1%, Sens: 60.0%, Spec: 75.0%, Prec: 36.8%
Neurological complications							
Chen, 2023	Cerebral complications	RF	Hold-out	0.828 (0.585, 0.902)	Brie*r* = 0.139	NI	Acc: 88.4%, Sens: 83.3%, Spec: 89.2%
Zhao, 2021	Pre-operation AIS	DNN	Hold-out	0.964 (0.932, 0.997)	NI	NI	Acc: 92.1%, Sens: 96.0%, Spec: 90.6%
Gastrointestinal bleeding							
Li, 2025	postoperative GIB	RF	5-fold CV	0.933 (0.840, 0.997)	Good calibration curve fitting	NI	NI
Composite outcome							
Lu, 2024	Postoperative adverse outcomes	XGBoost	Hold-out + External	0.985 (0.965, 1.000)	Good calibration curve fitting	NI	Acc: 92.0%, Sens: 94.0%, Spec: 91.0%, Prec: 92.0%
Luo, 2025	Major adverse outcomes	RSF + GBM	Hold-out	0.882	Brie*r* = 0.138	NI	NI
Pei, 2021	MAEs	LR	Hold-out	0.766 (0.718, 0.734)	NI	NRI = 0.653; IDI = 0.654	Acc: 77.0%, Prec: 65.3%
Wang, 2023	Adverse aortic events	CART	10-fold CV	0.852	Good calibration curve fitting; HL *P* = 0.586	NI	Acc: 88.9%, Sens: 77.8%, Spec: 92.6%
Xie, 2024	Postoperative adverse outcomes	XGBoost	10-fold CV	0.716 (0.668, 0.854)	Brie*r* = 0.192; Good calibration curve fitting	NI	NI
Xie, 2024	MAEs	RF	10-fold CV	0.975 (0.940, 1.000)	Brie*r* = 0.078; Good calibration curve fitting	NI	Acc: 88.7%
Other specified complications							
Chen, 2021	LOS	RF	5-fold CV	0.837 (0.766, 0.908)	NI	NI	NI
Dong, 2021	reintervention	LR	5-fold CV + Bootstrap	0.802 (0.689, 0.915)	Good calibration curve fitting	NI	Acc: 78.1%, Sens: 75.0%, Spec: 82.5%
Jin, 2025	MMP	RF	5-fold CV	0.797 (0.794, 0.800)	Brie*r* = 0.133; Good calibration curve fitting	NI	Acc: 74.0%, Sens: 81.1%, Spec: 72.2%
Jin, 2025	MMP	Deep learning model	5-fold CV + External	0.780 (0.777, 0.785)	Brie*r* = 0.143	NI	Acc: 76.0%, Sens: 66.7%, Spec: 78.3%
Li, 2025	Postoperative CRRT	XGBoost	5-fold CV	0.960 (0.930, 0.990)	Good calibration curve fitting	NI	Acc: 96.0%, Sens: 93.0%, Spec: 96.0%
Li, 2025	Prolonged LOS (exceeding 30 days)	XGBoost	Hold-out	0.710 (0.620, 0.800)	Good calibration curve fitting	NI	Acc: 71.0%, Sens: 76.0%, Spec: 84.0%
Lin, 2023	Death due to the dissection rupturing within 72 h after the CTA	CNN	Bootstrap	0.990 (0.950, 0.990)	Good calibration curve fitting; HL *P* = 0.91	NI	Acc: 90.0%, Spec: 90.0%, Prec: 90.0%
Ma, 2024	AGI	RF	NI	0.990 (0.966, 1.000)	NI	NI	NI
Pang, 2024	Complicated TBAD	LR	Hold-out	0.818 (0.641, 0.995)	NI	NI	NI
Wang, 2023	MS-ARDS within 24 h	LR	Hold-out	0.797	NI	NI	Acc: 80.0%, Prec: 66.3%
Wei, 2025	postoperative pulmonary complications	LightGBM	10-fold CV	0.763 (0.667, 0.859)	HL *P* = 0.32	NI	Acc: 75.3%, Sens: 65.0%, Spec: 82.0%, Prec: 70.3%
Wen, 2025	Postoperative reintubation	XGBoost	10-fold CV	0.969	Good calibration plots fitting	NI	Acc: 86.5%, Sens: 88.9%, Spec: 86.1%, Prec: 49.0%

AUC, area under the curve; ANN, Artificial Neural Network; BSS, Best Subset Selection; BPNN, Back Propagation Neural Networks; CART, Classification and Regression Trees; CNN, Convolutional Neural Network; CNB, Categorical Naive Bayes; DT, Decision Tree; DNN, deep neural network; ELM, Extreme Learning Machine; GBDT, Gradient Boosting Decision Tree; GBM, Gradient Boosting Machine; GNB, Gaussian Naive Bayes; IDI: integrated discrimination improvement; KNN, K-Nearest Neighbors; K-SVM, Kernel Support Vector Machine; LASSO, Least Absolute Shrinkage and Selection Operator; LR: logistic regression;LightGBM, Light Gradient Boosting Machine; MLP, Multilayer Perceptron; MLR, Multiple Logistic Regression; NB, Naive Bayes; NI, Not Provided; NN, Neural Network; NNET, Neural Network; NRI, Net Reclassification Improvement; PCA, Principal Component Analysis; PMM, Predictive Mean Matching; PSO, Particle Swarm Optimization; RF, Random Forest; RFE, Recursive Feature Elimination; RSF, Random Survival Forest; Ridge, Ridge Regression; SL, Super Learner; SVM, Support Vector Machine; SVC, Support Vector Classifier; SuperPC, Supervised Principal Components; XGBoost, Extreme Gradient Boosting.

#### 3.4.1 Early mortality (in-hospital and 30-day mortality)

**Figure 3:**
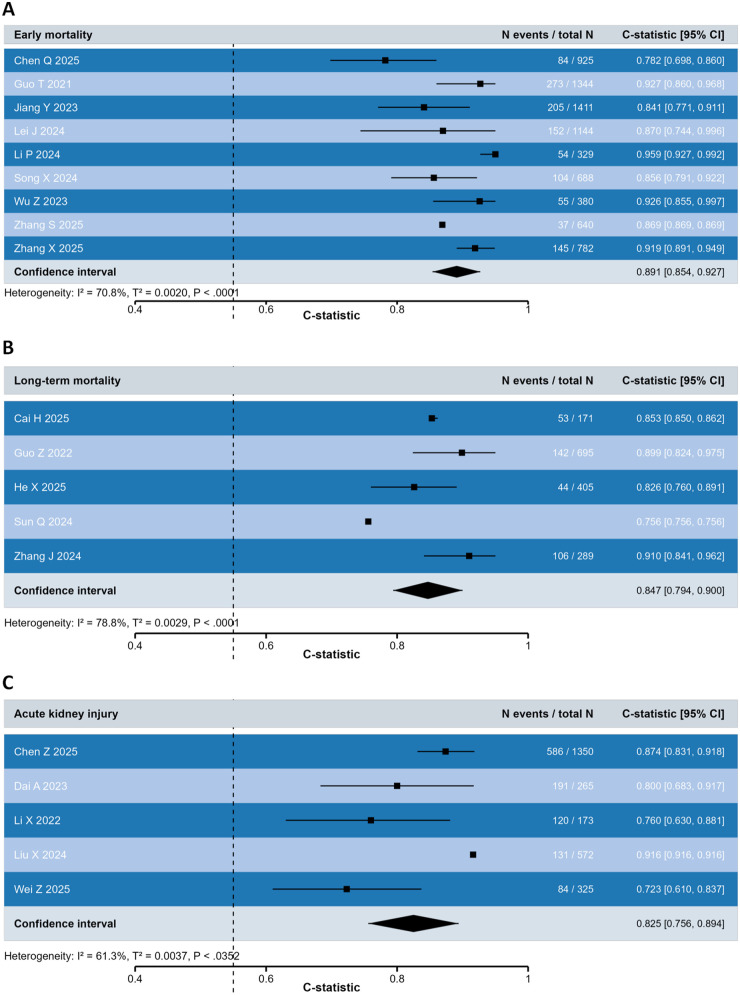
Meta-analysis of predictive performance of machine-learning models. Forest plot of outcome specific C-statistic (all 95% CIs estimated on the logit scale) stratified according to outcomes representing **(A)** early mortality, **(B)** long-term mortality, and **(C)** acute kidney injury. CI, confidence interval.

Nine studies developed machine learning models for predicting early mortality (in-hospital and 30-day mortality) in aortic dissection patients. From these studies, the top-performing prediction models were extracted from each study and included in our meta-analysis, comprising a diverse set of machine learning approaches including 3 XGBoost, 2 random forest, 1 bootstrap sampling strategy (BSS), 1 super learner (SL), 1 LR and 1 particle swarm optimization-extreme learning machine-flexible XGBoost (PSO-ELM-FLXGBoost) model. The meta-analysis demonstrated excellent overall predictive performance for early mortality prediction in aortic dissection patients, with a pooled C-statistic of 0.891 (95% CI: 0.854–0.927). A random-effects model was adopted due to significant heterogeneity among studies (*I*^2^ = 70.8%, *P* < 0.0001). However, due to this substantial heterogeneity, the pooled estimate should be interpreted as the average performance across a diverse set of studies. The 95% prediction interval for the C-statistic ranged from 0.669 to 0.972, indicating that while the average performance is high, the true underlying C-statistic in an individual (future) study could plausibly fall anywhere within this wide range (Figure 3A). Individual study performance ranged from 0.782 [(Chen, 2025) to 0.959 (Li, 2024)], indicating consistently strong discrimination ability across different models and patient populations. Among the included studies, Li, 2024 reported the highest predictive performance (C-statistic: 0.959; 95% CI: 0.927–0.992) using a super learner approach, while Guo, 2021 (C-statistic: 0.927; 95% CI: 0.860–0.968) and Wu, 2023 (C-statistic: 0.926; 95% CI: 0.855–0.997) also demonstrated outstanding performance using XGBoost algorithms. The study sample sizes varied substantially, with event rates ranging from 5.8% (37/640, Zhang, 2025) to 16.4% (54/329, Li, 2024), reflecting the heterogeneity in patient populations across different healthcare settings.

As detailed in Table 4, the machine learning models for early mortality prediction demonstrated robust performance across various evaluation metrics. The XGBoost model by Guo, 2021 achieved outstanding accuracy of 91.8% with high precision (68.3%), while maintaining excellent discriminative ability (AUC: 0.927). Similarly, Li, 2024's SL model exhibited remarkable sensitivity of 98.9% with specificity of 75.9%, achieving near-perfect AUC of 0.959 with the best calibration among all models (Brier score: 0.046). The ensemble approaches showed particularly strong performance, with Zhang, 2025's XGBoost model demonstrating balanced metrics including 90.1% accuracy, 81.4% sensitivity, 92.1% specificity, 70.6% precision, and AUC of 0.941. Notably, several studies reported reclassification metrics, with Pei, 2021's logistic regression model showing substantial net reclassification improvement (NRI = 0.654) and integrated discrimination improvement (IDI = 0.136), while Chen, 2025's SVM model demonstrated more modest improvements (NRI = 0.143, IDI = 0.062). The Brier scores across studies ranged from 0.046 to 0.058, indicating generally good calibration performance, with Li, 2024's model showing exceptional calibration. The consistent high performance across multiple evaluation metrics underscores the clinical utility of these machine learning models for early risk stratification and intervention planning in aortic dissection management.

##### 3.4.1.1 Sensitivity analysis and publication bias assessment

The sensitivity analysis using the leave-one-out method revealed that the pooled C-statistic ranged from 0.879 to 0.902, indicating good robustness of our primary finding. The exclusion of Chen, 2025 yielded the highest pooled estimate (C-statistic = 0.902, 95% CI: 0.871–0.933), while the exclusion of Li, 2024 resulted in the lowest estimate (C-statistic = 0.880, 95% CI: 0.848–0.912) (Supplementary Figure S1A).

Notably, the exclusion of Li, 2024 demonstrated the most substantial reduction in heterogeneity (*I*^2^ = 57.8% vs. 70.8% in the primary analysis), suggesting this study contributed significantly to the observed heterogeneity. Similarly, the exclusion of Chen, 2025 also substantially reduced heterogeneity (*I*^2^ = 58.9%). However, considerable heterogeneity persisted across most sensitivity analyses (*I*^2^ range: 57.8%–74.3%), indicating that the heterogeneity was not driven solely by any single outlier study (Supplementary Figure S2A).

The methodological sensitivity analysis comparing different meta-analytical approaches (REML, DL, HE, HS, and SJ) showed highly consistent pooled estimates ranging from 0.890 to 0.892, further supporting the robustness of our findings. Assessment of publication bias using Egger's test revealed no significant funnel plot asymmetry (*z* = −1.4117, *P* = 0.1580) (Supplementary Figure S3A). The symmetrical distribution of studies in the funnel plot suggests that there is no strong evidence of small-study effects or publication bias for the early mortality outcome. This finding enhances our confidence that the pooled estimate, despite the high heterogeneity, is not systematically inflated by the preferential publication of small, positive studies.

##### 3.4.1.2 Subgroup analysis

Subgroup analyses were performed to explore potential sources of heterogeneity among machine-learning models developed for early mortality prediction in patients with aortic dissection (Supplementary Table S7). Models derived from acute aortic dissection (AAD) cohorts (*n* = 5) demonstrated moderate heterogeneity (*I*^2^ = 73.5%, *P* = 0.005), with a pooled C-statistic of 0.87 (95% CI: 0.79–0.92) under a random-effects model. In contrast, models focusing specifically on acute type A aortic dissection (ATAAD) populations (*n* = 3) showed no evidence of heterogeneity (*I*^2^ = 0%, *P* = 0.968) and achieved a pooled C-statistic of 0.92 (95% CI: 0.89–0.94). Only one study was based on the broader “AD” category, thus meta-analysis was not applicable.

Studies with sample sizes ≥1,000 (*n* = 3) exhibited significant heterogeneity (*I*^2^ = 100%, *P* < 0.001), yielding a pooled C-statistic of 0.89 (95% CI: 0.82–0.93). Similarly, studies with sample sizes <1,000 (*n* = 6) showed substantial heterogeneity (*I*^2^ = 71.2%, *P* = 0.004) and had a pooled performance of 0.89 (95% CI: 0.80–0.94). Overall, model performance appeared consistent regardless of sample-size thresholds.

Models developed with EPV <10 (*n* = 3) showed extreme heterogeneity (*I*^2^ = 100%, *P* < 0.001) and a pooled C-statistic of 1.00 (95% CI: 0.82–0.96), although this estimate was unstable due to very high between-study variability. Models with EPV between 10 and 20 (*n* = 4) also demonstrated substantial heterogeneity (*I*^2^ = 100%, *P* < 0.001), with a pooled performance of 0.90 (95% CI: 0.83–0.94). Models with EPV > 20 (*n* = 2) showed slightly lower heterogeneity (*I*^2^ = 97.2%, *P* < 0.001) and achieved a pooled C-statistic of 0.85 (95% CI: 0.83–0.86).

Subgrouping based on modeling strategies revealed that models combining logistic regression with machine-learning algorithms (*n* = 7) had substantial heterogeneity (*I*^2^ = 77.6%, *P* < 0.001) and yielded a pooled C-statistic of 0.89 (95% CI: 0.84–0.95). Single-method subgroups including “LR only” and “ML models only” each contained one study; therefore, meta-analysis could not be performed for these categories.

Models validated using a hold-out approach (*n* = 4) demonstrated significant heterogeneity (*I*^2^ = 77.3%, *P* = 0.032) and produced a pooled C-statistic of 0.86 (95% CI: 0.78–0.94). Those validated using cross-validation (*n* = 2) showed comparable heterogeneity (*I*^2^ = 72.5%, *P* = 0.057) and achieved a pooled C-statistic of 0.89 (95% CI: 0.80–0.97). Interestingly, studies incorporating both hold-out and external validation (*n* = 2) had no detectable heterogeneity (*I*^2^ = 0%, *P* = 0.458) and yielded the highest pooled performance of 0.92 (95% CI: 0.89–0.94). Only one study lacked a clear validation description, precluding meta-analysis.

The subgroup analyses provided valuable insights into the sources of heterogeneity. Notably, models developed in ATAAD populations showed no heterogeneity (*I*^2^ = 0%) and a higher pooled C-statistic (0.92) compared to the broader AAD cohort (0.87, with high heterogeneity). This suggests that a more homogenous patient phenotype leads to more stable and potentially more accurate predictions.

Furthermore, the analysis by validation strategy was particularly revealing. Studies that included both hold-out and external validation demonstrated no heterogeneity (*I*^2^ = 0%) and the highest pooled performance (C-statistic = 0.92). In contrast, studies relying solely on internal validation (hold-out or cross-validation) showed substantial heterogeneity. This finding strongly underscores the critical role of rigorous, multi-faceted validation in producing reliable and generalizable performance estimates, and suggests that the lack of external validation is a major contributor to the overall heterogeneity we observed.

#### 3.4.2 Long-term mortality

Five studies developed machine learning models for predicting long-term mortality in aortic dissection patients. From these studies, the top-performing prediction models were extracted from each study and included in our meta-analysis, comprising diverse machine learning approaches including 1 neural network, 1 random survival forest with backward selection [StepCox(backward) + RSF], 1 random survival forest (RSF), 1 treebag, and 1 SVM model. The meta-analysis demonstrated excellent overall predictive performance for long-term mortality prediction in aortic dissection patients, with a pooled C-statistic of 0.847 (95% CI: 0.794–0.900), albeit with significant heterogeneity (*I*^2^ = 78.8%, *P* < 0.0001). The 95% prediction interval ranged from 0.530 to 0.969, highlighting the considerable uncertainty and variability in predictive performance across different settings and populations (Figure 3B). Individual study performance showed considerable variation, ranging from 0.756 (Sun, 2024) to 0.910 (Zhang, 2024), reflecting the heterogeneity in follow-up durations and patient populations across different studies. Among the included studies, Zhang, 2024 reported the highest predictive performance (C-statistic: 0.910; 95% CI: 0.841–0.962) using a treebag algorithm, closely followed by Guo, 2022 (C-statistic: 0.899; 95% CI: 0.824–0.975) employing a neural network approach. The study by Cai, 2025 also demonstrated strong performance (C-statistic: 0.853; 95% CI: 0.850–0.862) with an SVM model. The event rates varied across studies, with Cai, 2025 reporting 31.0% (53/171) mortality and Zhang, 2024 reporting 36.7% (106/289) mortality during their respective follow-up periods.

As detailed in Table 4, the long-term mortality prediction models exhibited robust performance across various evaluation metrics. The neural network model by Guo, 2022 achieved an accuracy of 87.1% with balanced sensitivity (65.0%) and specificity (92.3%), along with a Brier score of 0.120 indicating good calibration. Similarly, Zhang, 2024's treebag model demonstrated strong performance with 81.6% accuracy and a Brier score of 0.128. The random survival forest model by Sun, 2024, while showing the lowest C-statistic among the long-term mortality studies (0.756), maintained reasonable calibration with a Brier score of 0.137. Cai, 2025's SVM model achieved high accuracy (87.7%) and precision (82.4%) with a Brier score of 0.132, indicating consistent performance across different metrics. Notably, the machine learning approaches for long-term mortality prediction demonstrated performance comparable to, and in some cases superior to, the early mortality prediction models, suggesting their potential utility in prognostic assessment and long-term management planning for aortic dissection survivors. The variation in follow-up durations and outcome definitions across studies highlights the need for standardized outcome measures in future research on long-term prognosis in aortic dissection patients.

##### 3.4.2.1 Sensitivity analysis and publication bias assessment

The sensitivity analysis using the leave-one-out method revealed that the pooled C-statistic ranged from 0.829 to 0.865, indicating acceptable robustness of our primary finding. Specifically, the exclusion of Sun, 2024 yielded the highest pooled estimate (C-statistic = 0.865, 95% CI: 0.834–0.896), while the exclusion of Zhang, 2024 resulted in the lowest estimate (C-statistic = 0.829, 95% CI: 0.770–0.888) (Supplementary Figure S1B).

Notably, the exclusion of Sun, 2024 demonstrated the most substantial reduction in heterogeneity (*I*^2^ = 44.9% vs. 78.8% in the primary analysis), suggesting this study contributed significantly to the observed heterogeneity. However, considerable heterogeneity persisted in most sensitivity analyses (*I*^2^ range: 44.9%–83.6%), indicating that the heterogeneity was not driven solely by any single outlier study (Supplementary Figure S2B). This persistent heterogeneity reinforces that the variability in long-term mortality prediction is likely attributable to multiple factors, including differences in follow-up durations, outcome definitions, and patient case-mix across studies, rather than the influence of a single anomalous dataset.

The methodological sensitivity analysis comparing different meta-analytical approaches (REML, DL, HE, and HS) showed consistent pooled estimates ranging from 0.834 to 0.844, further supporting the robustness of our findings. Assessment of publication bias using Egger's test revealed no significant funnel plot asymmetry (*z* = 0.8588, *P* = 0.3905) (Supplementary Figure S3B). The symmetrical distribution of studies in the funnel plot suggests an absence of significant small-study effects for the long-term mortality outcome. This finding implies that the pooled estimate is unlikely to be substantially inflated by the preferential publication of smaller studies with overly optimistic results, lending some credence to the observed predictive performance despite the high heterogeneity.

##### 3.4.2.2 Subgroup analysis

Subgroup analyses were conducted to investigate potential sources of heterogeneity across machine-learning models developed to predict long-term mortality in patients with aortic dissection (Supplementary Table S8). Models developed in type A aortic dissection (TAAD) populations (*n* = 2) demonstrated considerable heterogeneity (*I*^2^ = 70.4%, *p* = 0.0661), yielding a pooled C-statistic of 0.87 (95% CI: 0.82–0.93) under a random-effects model. Models based on broader AAD cohorts (*n* = 2) showed moderate heterogeneity (*I*^2^ = 40.8%, *p* = 0.1940) and demonstrated comparable predictive performance, with a pooled C-statistic of 0.86 (95% CI: 0.77–0.91). Only one study focused specifically on ATAAD; therefore, meta-analysis was not applicable. The small number of studies within each subgroup limits definitive conclusions, but the persistent heterogeneity even within specific population types (e.g., TAAD) suggests that factors beyond simple anatomical classification, such as treatment protocols or follow-up duration, are major drivers of variability.

Studies with sample sizes ≥500 (*n* = 2) showed substantial heterogeneity (*I*^2^ = 81.1%, *P* = 0.0220), resulting in a pooled C-statistic of 0.83 (95% CI: 0.64–0.93). In contrast, models trained on sample sizes <500 (*n* = 3) demonstrated no observable heterogeneity (*I*^2^ = 0%, *P* = 0.2326) and achieved a pooled C-statistic of 0.85 (95% CI: 0.85–0.86), indicating more stable but slightly lower discriminatory ability. This pattern is somewhat counterintuitive, as larger samples are typically expected to yield more stable estimates. The high heterogeneity in the larger-sample subgroup may reflect greater diversity in patient populations or clinical settings in those studies, which, while increasing generalizability, also introduces variability that smaller, more homogenous, single-center studies lack.

Models developed with EPV <10 (*n* = 2) were homogeneous (*I*^2^ = 0%, *P* = 0.3904) and produced a pooled C-statistic of 0.85 (95% CI: 0.85–0.86). Similarly, models with EPV between 10 and 20 (*n* = 2) also exhibited no heterogeneity (*I*^2^ = 0%, *P* = 0.8223), showing higher pooled performance with a C-statistic of 0.91 (95% CI: 0.85–0.94). One study did not report EPV, preventing meta-analytic pooling. The lack of heterogeneity and the higher point estimate in the EPV 10–20 group suggests that while a minimum EPV is necessary to avoid overfitting, simply having a very high EPV (in the <10 group, the lack of heterogeneity may be due to uniformly poor model development) does not guarantee better or more consistent performance. However, these findings should be interpreted cautiously given the very small number of studies in each stratum.

Models validated using a hold-out approach (*n* = 2) were homogeneous (*I*^2^ = 0%, *P* = 1.0000) with a pooled C-statistic of 0.83 (95% CI: 0.76–0.89). Those utilizing cross-validation (*n* = 2) also demonstrated no heterogeneity (*I*^2^ = 0%, *P* = 0.8237) and achieved superior pooled performance, with a C-statistic of 0.91 (95% CI: 0.86–0.95). Only one model incorporated external validation, precluding further pooled analysis. The apparent superiority of cross-validation over simple hold-out validation in this subgroup analysis is notable, as cross-validation typically provides a more robust and less optimistic estimate of internal performance by utilizing the data more efficiently. The absence of a pooled analysis for externally validated models in this outcome category is a critical gap, as it leaves the true generalizability of these models unknown.

#### 3.4.3 Acute kidney injury

Five studies developed machine learning models for predicting acute kidney injury (AKI) in aortic dissection patients. From these studies, we extracted the top-performing prediction models for meta-analysis, which encompassed a range of machine learning approaches including 1 LightGBM models, 1 XGBoost, 1 random forest, 1 artificial neural network (ANN), and 1 CatBoost approach. The meta-analysis revealed strong overall predictive performance for AKI prediction in aortic dissection patients, with a pooled C-statistic of 0.825 (95% CI: 0.756–0.894). Reflecting the substantial heterogeneity (*I*^2^ = 61.3%, *P* = 0.035), the 95% prediction interval was wide, ranging from 0.384 to 0.974 (Figure 3C). Individual study performance varied considerably, ranging from 0.723 (Wei, 2025) to 0.916 (Liu, 2024), reflecting differences in patient populations, AKI definitions, and model architectures across studies. Among the included studies, Liu, 2024 demonstrated the highest predictive performance (C-statistic: 0.916; 95% CI: 0.916–0.916) using an artificial neural network approach, followed closely by Chen, 2025 (C-statistic: 0.874; 95% CI: 0.831–0.918) employing a LightGBM algorithm. Dai, 2023 also showed good performance (C-statistic: 0.800; 95% CI: 0.683–0.917) with an XGBoost model. The event rates varied across studies, with Dai, 2023 reporting the highest AKI incidence at 72.1% (191/265), while Wei, 2025 reported the lowest incidence at 25.8% (84/325).

As detailed in Table 4, the AKI prediction models exhibited variable performance across different evaluation metrics. The LightGBM model by Chen, 2025 demonstrated excellent discriminative ability without reported accuracy metrics, while Wei, 2025's CatBoost model achieved moderate accuracy (72.1%) with balanced sensitivity (60.0%) and specificity (75.0%), though with relatively low precision (36.8%). Liu, 2024's ANN model demonstrated high sensitivity (94.0%) and specificity (81.0%), contributing to its superior discriminative performance. Notably, the machine learning approaches for AKI prediction showed promising results despite the clinical complexity of this complication in aortic dissection patients. The substantial heterogeneity observed in our analysis likely stems from variations in AKI definitions, timing of assessment, and patient characteristics across different healthcare settings. These findings suggest that machine learning models have potential utility in identifying patients at high risk for this serious complication, though standardization of outcome definitions and feature selection would enhance comparability across future studies.

##### 3.4.3.1 Sensitivity analysis and publication bias assessment

Sensitivity analysis using the leave-one-out method revealed that the pooled C-statistic estimates ranged from 0.803 to 0.859, demonstrating moderate robustness of the primary finding. The exclusion of Wei, 2025 yielded the highest pooled estimate (C-statistic = 0.859, 95% CI: 0.795–0.923), while the exclusion of Liu, 2024 resulted in the lowest estimate (C-statistic = 0.803, 95% CI: 0.728–0.879) (Supplementary Figure S1C).

Notably, the exclusion of Wei, 2025 demonstrated the most substantial reduction in heterogeneity (*I*^2^ = 40.3% vs. 61.3% in the primary analysis), suggesting this study contributed significantly to the observed between-study variability. The methodological sensitivity analysis comparing different meta-analytical approaches showed consistent pooled estimates ranging from 0.831 to 0.851 across REML, DL, HE, and HS methods (Supplementary Figure S2C).

Assessment of publication bias using Egger's test revealed significant funnel plot asymmetry (*z* = −4.3394, *P* < 0.0001), indicating potential small-study effects or other sources of bias in the literature (Supplementary Figure S3C). This significant publication bias suggests that smaller studies with positive or higher-performing results may be overrepresented in the literature for AKI prediction. Consequently, our pooled C-statistic of 0.825 is likely an overestimate of the true predictive performance of ML models for this outcome in real-world settings. This finding is a major limitation for the AKI analysis and suggests that the body of evidence is skewed. Readers should therefore place less confidence in the pooled estimate for AKI compared to the mortality outcomes, where no such bias was detected. The true performance of ML models for AKI prediction may be closer to the lower end of the confidence interval or prediction interval than the pooled mean suggests.

Individual study C-statistics varied considerably, ranging from 0.723 (Wei, 2025) to 0.916 (Liu, 2024), with sample sizes spanning from 173 (Li, 2022) to 1,350 (Chen, 2025) participants. This variability in both model performance and study characteristics likely contributed to the substantial heterogeneity observed in the meta-analysis.

##### 3.4.3.2 Subgroup analysis

Subgroup analyses were performed to explore the potential sources of heterogeneity in machine-learning models developed to predict AKI in patients with aortic dissection (Supplementary Table S9). Models developed in AAD cohorts (*n* = 2) showed no evidence of heterogeneity (*I*^2^ = 0%, *P* = 0.671) and demonstrated a pooled C-statistic of 0.74 (95% CI: 0.65–0.81). In contrast, models derived from ATAAD populations (*n* = 3) exhibited substantial heterogeneity (*I*^2^ = 76.8%, *P* = 0.020), with a higher pooled predictive performance of 0.88 (95% CI: 0.81–0.93). These findings suggest that population specificity may influence model performance and heterogeneity in AKI prediction. The higher performance and greater heterogeneity in the ATAAD subgroup may reflect the more complex and variable perioperative course of these patients compared to the broader AAD population, making prediction both more challenging (hence the heterogeneity) and potentially more rewarding (hence the higher AUC) if key factors are captured.

Subgroup analysis by sample size showed that only models developed with sample sizes <500 (*n* = 3) could be pooled, revealing no detectable heterogeneity (*I*^2^ = 0%, *P* = 0.662) and achieving a pooled C-statistic of 0.76 (95% CI: 0.68–0.82). Single-study subgroups (≥1,000 or 500–1,000) could not be synthesized, limiting further interpretation. The stability and lower performance of the smaller-sample subgroup may indicate that these models, while consistent, are underpowered to capture the full complexity of AKI prediction, or that they come from more homogenous populations.

Models developed with EPV between 10 and 20 (*n* = 2) demonstrated very high heterogeneity (*I*^2^ = 90.5%, *P* = 0.001) and produced a pooled C-statistic of 0.86 (95% CI: 0.65–0.95). Models with EPV >20 (*n* = 2) showed moderate heterogeneity (*I*^2^ = 40.7%, *P* = 0.194) and a pooled performance of 0.85 (95% CI: 0.78–0.91). Only one study reported EPV <10; thus, meta-analysis was not feasible for that subgroup. The high heterogeneity in the EPV 10–20 group, despite meeting a minimum sample size requirement, suggests that other methodological factors (e.g., predictor selection, handling of missing data) play a more dominant role in causing variability than EPV alone. The more stable (though still moderately heterogeneous) estimate in the EPV >20 group suggests that larger, well-powered studies may produce more consistent results.

Machine-learning models validated using cross-validation (*n* = 3) exhibited moderate heterogeneity (*I*^2^ = 67.8%, *P* = 0.035) and demonstrated a pooled C-statistic of 0.81 (95% CI: 0.72–0.90). Models validated through bootstrap or mixed approaches (cross-validation + bootstrap) were each represented by a single study, preventing quantitative synthesis. The reliance on internal validation methods only (cross-validation) for all pooled studies in this analysis, combined with the significant publication bias detected, highlights the critical need for externally validated AKI prediction models to determine their true clinical utility.

#### 3.4.4 Other relevant outcomes

Several studies developed machine learning models for predicting other clinically important outcomes in aortic dissection patients, encompassing a range of composite endpoints and specific complications.

Eight studies developed machine learning models for predicting composite outcomes in patients with aortic dissection, encompassing a spectrum of clinically relevant endpoints including major adverse events, mortality and aortic-related events, and postoperative adverse outcomes. Wang Y, 2023 utilized a CART algorithm to predict adverse aortic events, achieving a C-statistic of 0.852 with balanced sensitivity (77.8%) and specificity (92.6%). Pei, 2021 focused on major adverse events (MAEs) using logistic regression, demonstrating robust performance with a C-statistic of 0.776, accuracy of 77.0%, precision of 65.3%, and significant net reclassification improvement (NRI: 0.654) and integrated discrimination improvement (IDI: 0.136). Pang, 2024 employed multiple algorithms including LR, SVM, KNN, RF, DT, and XGBoost to predict complicated type B aortic dissection. Their best-performing model achieved a C-statistic of 0.818 in the validation cohort, with the development cohort reaching 0.877. He, 2025 developed a StepCox[backward] + RSF model to predict a composite endpoint of mortality and aortic-related events, demonstrating good discriminative ability with a C-statistic of 0.826. Two studies by Xie, 2024 addressed different composite outcomes. The first developed multiple models for postoperative adverse outcomes, with XGBoost achieving a C-statistic of 0.716 and RF reaching 0.975. The second study focused on MAEs in malnourished aortic dissection patients, where RF demonstrated excellent performance (C-statistic: 0.975) with good calibration (Brier score: 0.078). Luo, 2025 employed an ensemble approach (RSF + GBM) to predict major adverse outcomes, achieving strong discriminative performance (C-statistic: 0.882) with satisfactory calibration (Brier score: 0.138). Lu, 2024 investigated postoperative adverse outcomes in uncomplicated type B aortic dissection using XGBoost, demonstrating exceptional predictive performance with C-statistics of 0.985–0.990 across different validation sets, along with high accuracy (92.0%), sensitivity (94.0%), specificity (91.0%), and precision (92.0%).

Procedure-specific complications were addressed in multiple studies. Li, 2025 developed a random forest model to predict postoperative gastrointestinal bleeding (GIB) in type A aortic dissection patients, showing excellent predictive performance with a C-statistic of 0.933. For pulmonary complications, Wei, 2025 employed multiple machine learning approaches, with the best-performing model (specific algorithm not reported in available data) achieving a C-statistic of 0.763. Wen, 2025 focused on predicting postoperative reintubation using XGBoost, which demonstrated outstanding discriminative ability with a C-statistic of 0.969 and balanced sensitivity (88.9%) and specificity (86.1%), though with moderate precision (49.0%).

Two studies developed machine learning models for predicting neurological complications in aortic dissection patients, addressing distinct but clinically significant central nervous system outcomes. Zhao H, 2021 focused on predicting pre-operation acute ischemic stroke (AIS) in acute type A aortic dissection patients. The study employed a comprehensive modeling approach including SVM, RF, NN, and DNN algorithms. The deep neural network (DNN) model demonstrated the best performance with an exceptional C-statistic of 0.964 (95% CI: 0.932–0.997) in the validation cohort, along with high accuracy (92.1%), sensitivity (96.0%), and specificity (90.6%). The development cohort achieved even higher performance with a C-statistic of 0.982 (95% CI: 0.967–0.997). Chen H, 2023 developed prediction models for cerebral complications in acute type A aortic dissection patients, comparing multiple machine learning approaches including LR, KNN, RF, GBM, SVM, and MLP. The random forest algorithm emerged as the best-performing model, achieving a C-statistic of 0.828 (95% CI: 0.585–0.902) with balanced performance metrics including accuracy (88.4%), sensitivity (83.3%), and specificity (89.2%). The model also demonstrated good calibration with a Brier score of 0.139. Both studies demonstrated that machine learning approaches can effectively predict neurological complications in aortic dissection patients, though they addressed different neurological endpoints—pre-operative acute ischemic stroke and general cerebral complications. The consistently strong performance across both studies (C-statistics: 0.828–0.964) suggests that neurological complications represent a predictable endpoint in this patient population. The variation in model performance may reflect differences in outcome definitions, with the more specific outcome of acute ischemic stroke demonstrating higher predictive accuracy compared to the broader category of cerebral complications.

Other specific outcomes included mesenteric malperfusion predicted by Jin, 2025 using a random forest model (C-statistic: 0.797), postoperative continuous renal replacement therapy predicted by Li, 2025 using XGBoost (C-statistic: 0.960), and acute gastrointestinal injury predicted by Ma, 2024 using random forest (C-statistic: 0.990).

The performance metrics for these diverse outcomes demonstrated considerable variation, reflecting the different clinical challenges in predicting each specific complication. Models for some outcomes like postoperative CRRT and AGI showed exceptional discriminative ability (C-statistics >0.95), while others such as pulmonary complications and mesenteric malperfusion demonstrated more moderate but still clinically useful performance (C-statistics 0.76–0.80). This heterogeneity in predictive performance across different endpoints highlights the varying predictability of distinct complications in aortic dissection patients and suggests that machine learning approaches may be particularly valuable for certain specific clinical scenarios beyond the commonly studied mortality outcomes.

### 3.5 Risk of bias and applicability assessment

#### 3.5.1 Participants, predictors, outcomes

**Figure 4:**
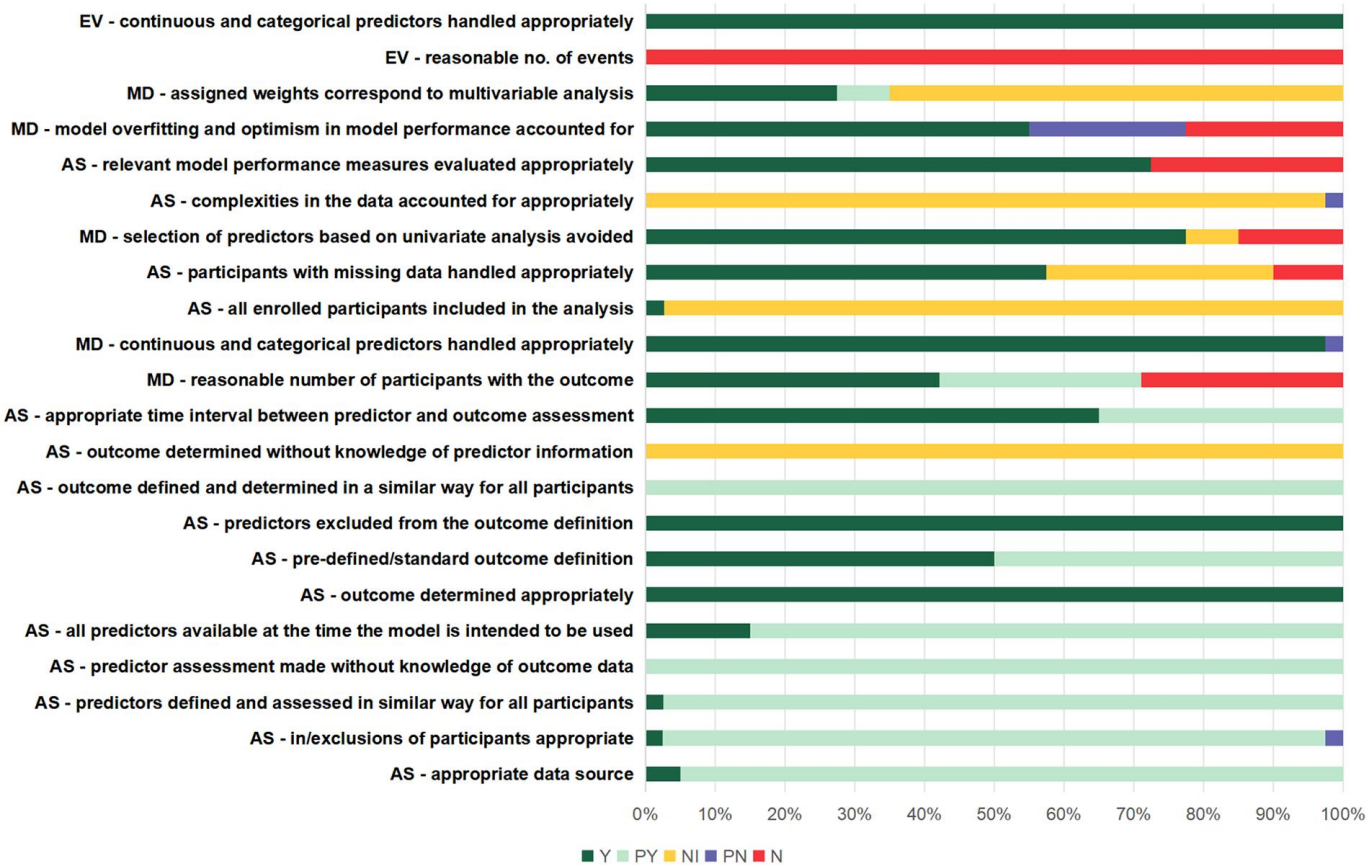
Risk of bias summary. Chart shows percentage of study cohorts meeting/not meeting criteria: AS, all studies (40 study cohorts): EV, external validation studies (7 study cohorts); ML, model development studies (40 study cohorts); N, no; NI, no or insufficient information: PN, probably no; PY, probably yes: Y, yes.

**Figure 5:**
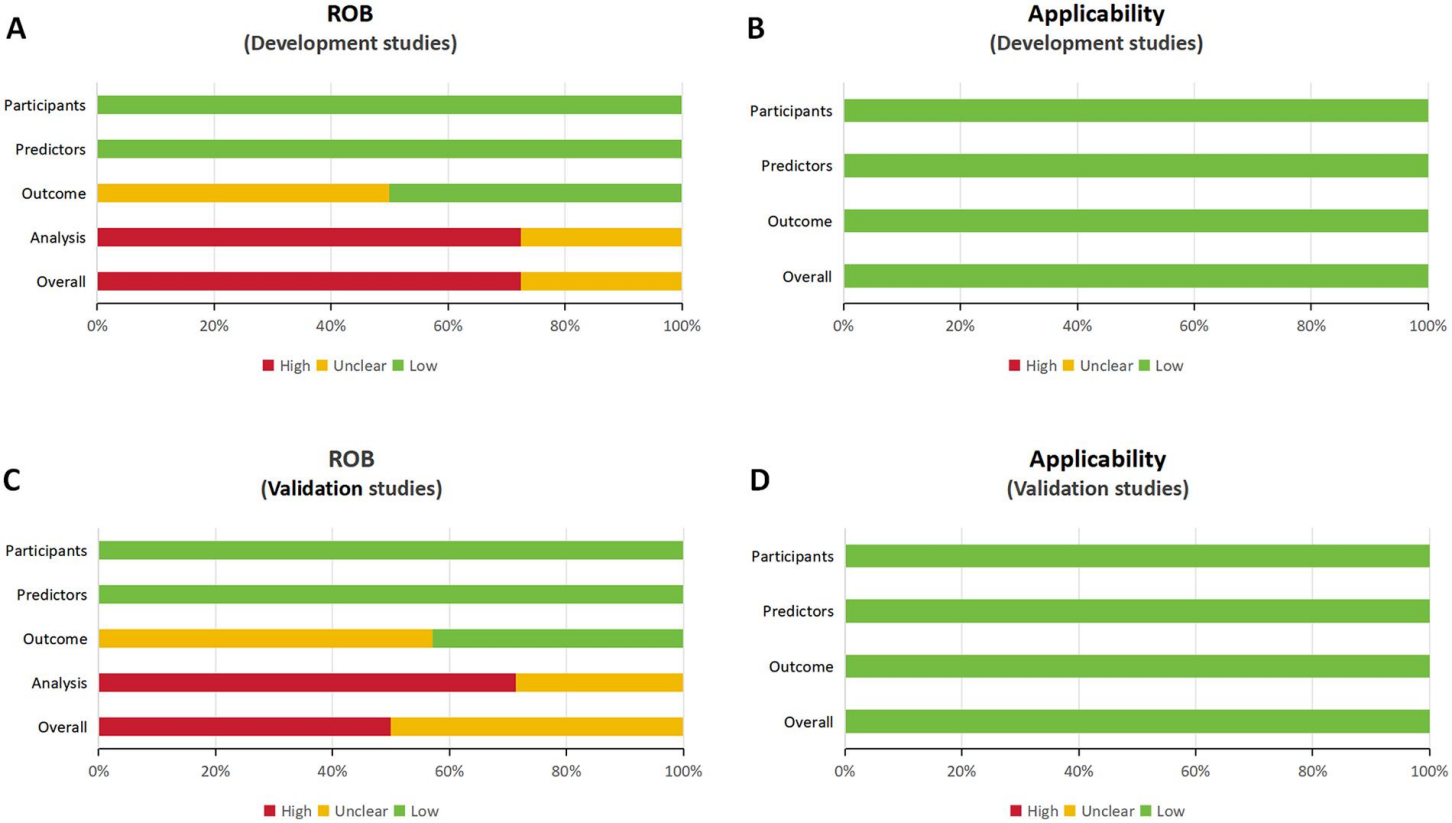
Prediction model risk of bias assessment tool (PROBAST) for the studies included in this review. **(A)** The risk of bias of the 40 development studies. **(B)** The applicability of the 40 development studies. **(C)** The risk of bias of the 7 validation studies. **(D)** The applicability of the 7 validation studies.

All 40 included studies were assessed for ROB and applicability concerns using the PROBAST tool. In the domain of participants, all studies were rated as having low ROB, as they utilized data sources appropriate for AD risk prediction, primarily from retrospective or prospective cohorts with clearly defined inclusion and exclusion criteria. However, 13 studies (32.5%) did not adequately report whether all enrolled participants were included in the final analysis or provided insufficient information on the handling of excluded cases, leading to potential selection bias. In the predictors domain, all studies were judged as low ROB, as predictors were generally defined and assessed in a consistent manner across participants, and most were measured at baseline prior to outcome occurrence. In the outcomes domain, 20 studies (50.0%) were rated as having unclear ROB, primarily due to insufficient reporting on whether outcome assessment was performed without knowledge of predictor information. Although outcomes such as in-hospital mortality or malperfusion were often based on objective criteria, the lack of blinding in outcome adjudication may introduce detection bias. A detailed PROBAST-based assessment of population, predictors, and outcomes are presented in Supplementary Tables S10 and S11 and are summarized visually in Figures 4, 5.

#### 3.5.2 Analysis-model development studies

Among the 40 studies, a proportion of the included studies (*n* = 11, 27.5%) did not meet the recommended threshold of at least 10 events per variable (EPV), with EPV values ranging from 4 to 61. This increases the risk of overfitting and limits the stability of model estimates. In terms of handling continuous predictors, 1 study (2.5%) were rated as having high ROB due to data-driven dichotomization (e.g., using ROC-derived cut-offs) without accounting for optimism. The remaining studies appropriately treated continuous variables as continuous or used pre-specified clinical thresholds. Missing data handling was inadequately reported in 13 development studies (32.5%). Among those that reported methods, multiple imputation was the most common approach (*n* = 13), while 4 studies excluded participants with missing data, potentially introducing bias. Variable selection methods also varied widely: 6 studies (15.0%) used univariate screening before multivariable modeling, which may lead to omitted predictors or inflated performance. In contrast, 31 studies (77.5%) used more robust methods such as LASSO, ridge regression, or machine learning-based feature selection. Internal validation was performed in 37 studies (92.5%), with cross-validation being the most common method (*n* = 19), followed by bootstrapping (*n* = 4) and hold-out validation (*n* = 16). Finally, 11 studies (27.5%) did not report both discrimination and calibration metrics, limiting comprehensive evaluation of model performance. The detailed PROBAST assessment of analysis-related bias is provided in Supplementary Tables S10 (analysis domain) and S11.

#### 3.5.3 Analysis-model evaluation studies

Of the 40 studies, only 7 (17.5%) included any form of external validation. However, the nature and robustness of this validation varied considerably. Five of these seven studies employed temporal validation, using patients treated more recently at the same institution. While better than no validation, temporal validation does not fully address generalizability, as it does not account for differences in patient demographics, clinical practices, or data acquisition protocols across different centers or healthcare systems. Only two studies (5.0%) performed validation on a truly independent cohort from a different hospital or region. All 7 studies were judged as having high ROB in the analysis domain, primarily due to small sample sizes or insufficient outcome events (<100 events) in the validation cohort, which may affect the reliability of performance estimates. All external validation studies applied the original model without retraining or recalibration, maintaining consistency with the development process. All seven studies that performed external validation employed identical procedures for handling continuous predictors as those used in the original model development. A summary of the overall risk of bias and applicability assessments is provided in Supplementary Table S11. The majority of studies were rated as having high or unclear overall ROB, primarily due to limitations in the analysis domain, whereas applicability concerns were generally low across all studies.

#### 3.5.4 Assessment of methodological issues specific to machine learning

Beyond the standard PROBAST framework, we conducted a deeper assessment of methodological issues specific to machine learning practices that could bias performance estimates.

First, a high risk of data leakage was pervasive. Our analysis of Table 3 revealed that in 25 studies (62.5%), critical steps such as feature selection (e.g., LASSO regression, univariate screening) or data imputation procedures (e.g., multiple imputation, KNN) were applied to the entire dataset before it was split into training and validation sets. This practice, identified in studies like Chen, 2025, Jiang, 2023, and Wei, 2025, potentially incorporates information from the test set into the model development process, leading to optimistic performance estimates. Furthermore, among the 32 studies (80.0%) that reported data normalization or scaling, the majority (*n* = 25, 78.1%) did not explicitly state that these parameters were calculated solely from the training set and then applied to the test set, raising concerns about the fairness of the evaluation.

Second, regarding validation strategies for time-dependent data, of the 15 studies with clear temporal components (e.g., long-term mortality follow-up as shown in Table 2 for studies like Guo, 2022, Sun, 2024, and Zhang, 2024), only 3 (20.0%) used time-series-specific validation methods (e.g., external temporal validation). The remaining 12 studies (80.0%) used random cross-validation, which is methodologically inappropriate for time-to-event data and may result in overly optimistic assessments by allowing the model to use “future” information to predict the past.

Third, the handling of class imbalance was inconsistent. 14 studies predicted outcomes with low event rates (<20%), such as Jin, 2025 (MMP, 1.6%), Zhang, 2025 (30-day mortality, 5.8%), and Xie, 2024 (in-hospital mortality, 5.8%). Among these, only 5 studies (35.7%) reported using techniques to address imbalance, such as reporting the Brier score for calibration or using specific algorithms robust to imbalance. The remaining studies relied solely on accuracy and AUC, which are known to be misleading performance metrics in the presence of imbalanced data, potentially explaining the discordance between high AUC and low precision in some cases (e.g., Wen, 2025 achieved an AUC of 0.969 but a precision of only 49.0%).

Finally, the risk of overfitting was not solely confined to low EPV. We observed that 10 studies (25.0%) applied highly complex algorithms, such as deep neural networks (DNN) or sophisticated ensemble methods (e.g., Sun, 2024's Deepsurv, Luo, 2025's multiple ensembles), on relatively small datasets (*n* < 300, as shown in Table 2 for studies like Chen, 2023, Li, 2024, and Ma, 2024). Without robust external validation or strong regularization, these models are highly susceptible to overfitting. This likely contributed to the inflated performance metrics and the wide 95% prediction intervals observed in our meta-analyses (e.g., 0.384–0.974 for AKI), indicating that model performance is highly unstable.

#### 3.5.5 Adherence to reporting standards

**Figure 6:**
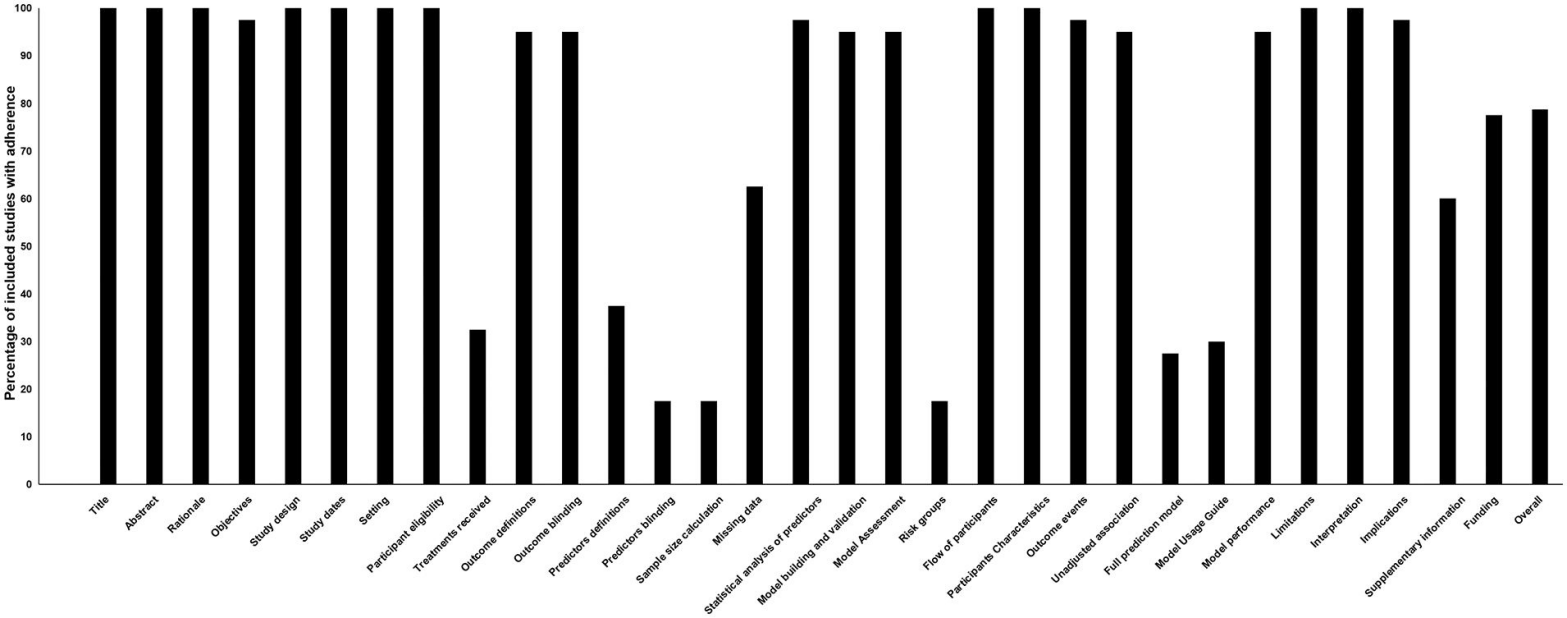
Adherence of included studies to transparent reporting of a multivariable preduction model for individual prognosis or diagnosis (TRIPOD) assessment.

Assessment of reporting quality using the TRIPOD checklist revealed an overall adherence rate of 78.7%, with considerable variation across individual checklist items. Essential elements such as title, abstract, rationale, study design, study dates, setting, participant eligibility, flow of participants, participants characteristics, limitations, and interpretation were consistently reported across all included studies (100%). Similarly, objectives, outcome definitions, outcome blinding, statistical analysis of predictors, model building and validation, model assessment, outcome events, unadjusted association, model performance, and implications were also highly reported, with adherence rates exceeding 95%. However, several critical methodological details were poorly reported. Only 32.5% of studies described treatments received, and merely 17.5% reported predictors blinding. Sample size calculation and risk groups were each reported in only 17.5% of studies. Predictors definitions were provided in 37.5% of studies, while full prediction model details were available in only 27.5%. Model usage guidance was provided in 30% of studies. Regarding data handling, missing data were addressed in 62.5% of studies. Supplementary Information was provided in 60% of studies, and funding was disclosed in 77.5%. In summary, while most studies adequately reported foundational and methodological elements, key aspects related to model transparency, blinding, sample size justification, and risk stratification were frequently neglected. Collectively, these deficiencies, quantified by the PROBAST and TRIPOD assessments, underscore a critical gap between model development and the prerequisites for clinical readiness. A detailed item-by-item adherence to the TRIPOD checklist is illustrated in Figure 6.

## 4 Discussion

This systematic review and meta-analysis provides a comprehensive and up-to-date evaluation of ML models developed for predicting adverse outcomes in patients with AD. Building upon prior prediction-modeling work in cardiovascular disease, this study synthesizes evidence across 40 studies that collectively explored a broad spectrum of clinical endpoints—including early mortality, long-term mortality, postoperative complications, AKI, neurological events, mesenteric ischemia, and composite outcomes. Our findings highlight both the substantial promise and the meaningful limitations of ML-based risk prediction in the management of AD.

**Figure 7:**
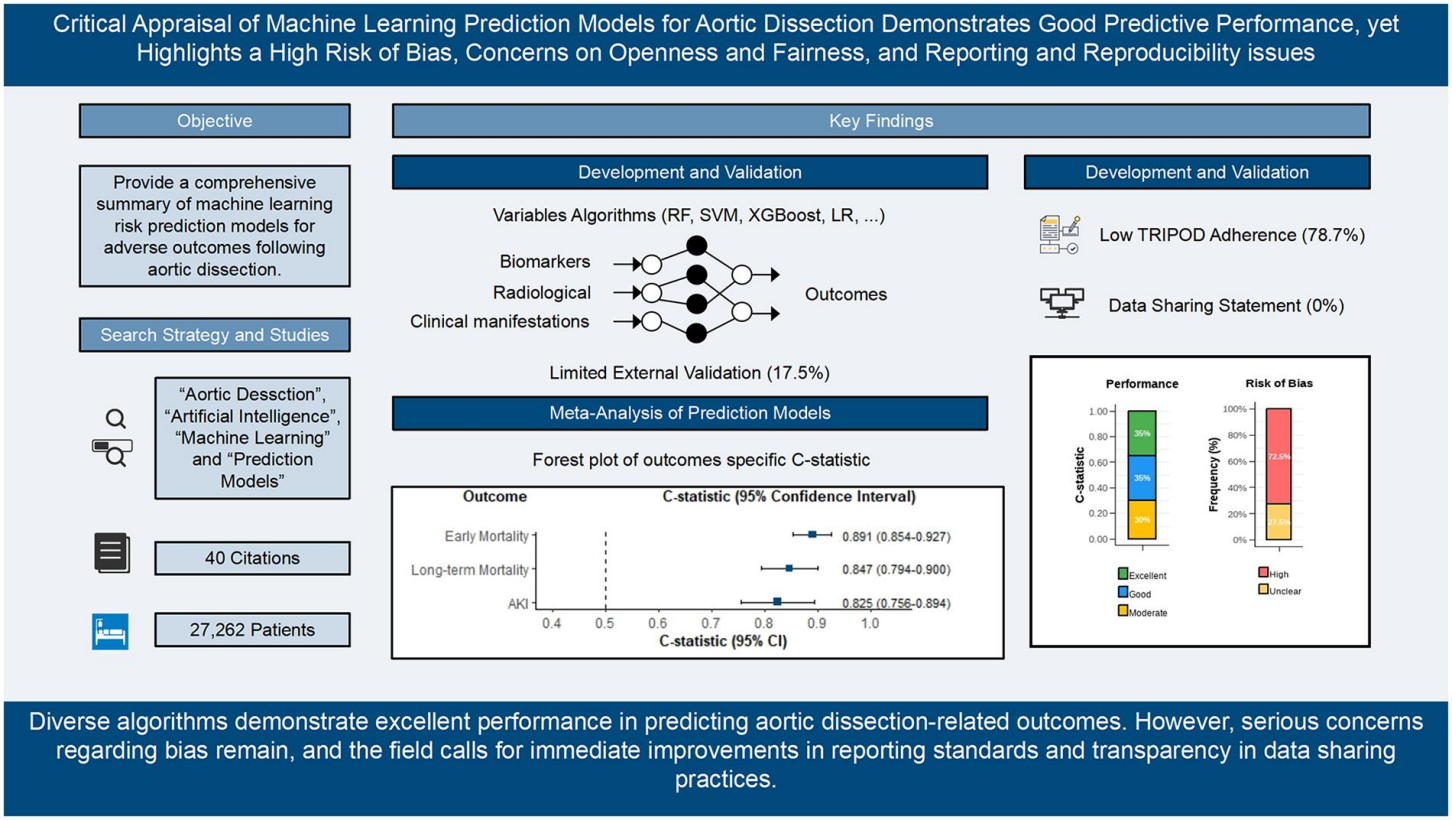
Visual summary of the main findings in our study. RF, random forest; SVM, support vector machine; XGBoost, extreme gradient boosting; LR, logistic regression; TRIPOD, transparent reporting of a multivariable prediction model for individual prognosis or diagnosis; Cl, confidence interval.

Notably, ML models demonstrated consistently strong discriminative ability across major outcomes. However, the methodological quality, reporting transparency, and robustness of validation approaches varied substantially across studies. These limitations currently impede the translation of ML models from research settings to real-world clinical practice. The following sections discuss the implications of our results in detail, explore possible explanations for observed heterogeneity, critically evaluate methodological challenges, and propose recommendations for future research (Figure 7).

### 4.1 Principal findings and interpretation

Our meta-analysis showed that ML models achieved excellent discriminative performance for early and long-term mortality, with pooled C-statistics of 0.891 and 0.847, respectively. These values surpass the typical performance of traditional logistic regression or Cox-based risk models reported in prior AD literature, which usually achieve AUC values between 0.70 and 0.82 (56–59). Importantly, this trend was consistent across studies using advanced algorithms such as XGBoost, RF, LightGBM, DNNs, and ensemble frameworks. However, these summary estimates are misleading if presented without their crucial context: they are derived from a body of literature with significant methodological flaws.

Compared with earlier risk models (e.g., IRAD-based logistic regression), ML approaches appear better suited to handle the complex interactions among clinical, laboratory, imaging, and perioperative parameters encountered in AD (60). This capability is particularly adept at modeling nonlinear dose–response associations in parameters like creatinine and D-dimer, unraveling higher-order interactions between disparate feature sets such as anatomy and hemodynamics, and accounting for complex perioperative time dependencies as well as multicollinearity among correlated laboratory variables (61). Such capabilities are particularly relevant for AD, which presents with substantial pathophysiological heterogeneity and a rapidly evolving clinical course. The consistently high performance of ML approaches suggests that they may outperform traditional prediction models in identifying patients at imminent risk of complications. While this may suggest that ML is well-suited to capture the complex pathophysiology of AD, it is equally, if not more, likely that these values are substantially inflated due to underlying methodological issues such as overfitting (from low events-per-variable), optimistic internal validation, and selective reporting.

Our pooled analysis of AKI models revealed a strong overall predictive performance (C-statistic: 0.825). Considering the multifactorial pathogenesis of AKI in AD patients—ranging from renal malperfusion and systemic hypoperfusion to perioperative stress and inflammatory activation—this finding is clinically meaningful. Models predicting neurological complications, postoperative gastrointestinal bleeding, and other organ-specific complications also achieved high C-statistics (often >0.90). This reinforces the idea that ML methods are adept at identifying subtle patterns within complex physiological systems, potentially enabling earlier recognition of complications that are difficult to predict through conventional statistical approaches (62).

Notwithstanding their promising discriminative metrics, many models were developed under suboptimal conditions that may lead to artificially high performance estimates. The methodological robustness of the included studies was frequently compromised by several recurrent limitations. Key concerns included the widespread absence of external validation, inadequate EPV ratios, and suboptimal handling of missing data. Compounding these issues were frequent omissions in reporting model calibration, the use of inappropriate variable selection methods such as univariate screening, and a high risk of overfitting stemming from the development of models with extensive predictor sets on relatively small sample sizes. Therefore, the primary finding of this review is not that ML models are “excellent,” but rather that despite their theoretical promise and consistently reported high AUCs, the current evidence base is too immature to support any claims of clinical superiority or readiness. The models' true performance in real-world, diverse populations remains unknown due to the pervasive lack of rigorous external validation.

### 4.2 Comparative performance of machine learning and traditional statistical models

A critical objective of this review was to contrast the performance of ML models with that of traditional statistical approaches (e.g., logistic regression, Cox regression) across four key domains: discrimination, calibration, interpretability, and clinical applicability.

In terms of discrimination, our meta-analysis suggests that ML models may offer a marginal advantage. The pooled C-statistics for early mortality (0.891) and long-term mortality (0.847) in our study appear numerically higher than those typically reported for conventional risk scores in AD, which often range between 0.70 and 0.82. This finding supports the hypothesis that ML algorithms are better equipped to capture complex, non-linear interactions among high-dimensional clinical, laboratory, and imaging variables. However, as noted in Section 4.1, this apparent superiority must be tempered by the high risk of bias in the included studies. The performance gain may be, at least in part, an artifact of overfitting or optimistic internal validation, rather than a true reflection of algorithmic superiority.

The picture is less clear for calibration, a domain where traditional models typically excel due to their inherent structure and rigorous development frameworks. While 72.5% of the included ML studies assessed calibration, the methods used were heterogeneous. More importantly, the lack of a consistent comparator (e.g., a well-fitted logistic regression model) in most studies makes it impossible to determine if ML models provide better-calibrated risk estimates. In fact, the few studies that did compare both approaches (e.g., Pei, 2021, Chen, 2023) showed mixed results, with some ML models exhibiting similar or even worse calibration than their simpler counterparts. This suggests that the flexibility of ML can be a double-edged sword; without careful regularization and *post-hoc* calibration, they may produce overconfident and poorly calibrated predictions.

The most significant divergence lies in interpretability. Traditional regression models offer inherent transparency, with coefficients providing direct insight into the direction and magnitude of each predictor's effect. This “white-box” nature is crucial for building clinician trust and for hypothesis generation. In contrast, the majority of ML models reviewed here function as “black boxes.” Although some studies have recently incorporated *post-hoc* explainability tools like SHAP (e.g., Li, 2025, Li, 2025), these methods provide only local approximations of feature importance and do not fully elucidate the model's internal decision-making logic. This lack of intrinsic interpretability remains a major barrier to clinical adoption, as physicians are often reluctant to act upon recommendations from a system whose reasoning they cannot scrutinize.

Finally, regarding clinical applicability, traditional models have a distinct advantage in their simplicity of deployment. A logistic regression model can be easily translated into a paper-based nomogram or a simple scoring system, making it accessible in a wide range of clinical settings, including those with limited digital infrastructure. ML models, by contrast, often require integration into electronic medical records (EMRs) with real-time computational support, posing significant implementation challenges. Furthermore, a rigorous assessment of clinical utility, such as decision curve analysis (DCA), was performed in only a small minority of studies. Consequently, while many ML models demonstrate high theoretical performance, evidence that their use would lead to a net clinical benefit—by improving decision-making without causing harm—is almost entirely lacking. This stands in stark contrast to the established methodology for evaluating clinical tools, which emphasizes net benefit over mere predictive accuracy.

### 4.3 Sources of heterogeneity

The observed heterogeneity in predictive performance across studies was substantial (*I*^2^ = 61.3%–78.8%), and this is further underscored by the wide prediction intervals. These intervals indicate that the true discriminatory ability of ML models for AD outcomes varies considerably, and a single summary statistic cannot adequately capture this variability. Several key methodological and clinical factors likely contributed. The included studies exhibited heterogeneity in the patient populations, particularly in the types of aortic dissection investigated. This variation encompassed distinctions such as acute vs. chronic presentations, type A vs. type B dissections, uncomplicated vs. complicated status, and the treatment modality (surgical vs. medical management). Furthermore, differences in perioperative strategies and institutional expertise contributed to the clinical diversity across the studies. These variations influence both the baseline risk profile and the predictive utility of specific clinical variables. For example, renal malperfusion is a dominant predictor in type B AD but less relevant in type A cases undergoing emergent surgery. Such heterogeneity complicates direct comparison and pooling of results. In long-term mortality models, follow-up durations ranged from 1 to 3 years. For AKI, criteria varied across KDIGO, RIFLE, or institution-specific definitions. Composite outcomes were even more variable. These inconsistencies likely contributed to the observed heterogeneity and limited the interpretability of pooled results. Differences in model-building decisions—including missing data handling, feature selection (LASSO, univariate screening, recursive feature elimination), choice of ML algorithm, class imbalance correction, and validation strategy—represent major sources of variability. Studies employing external validation generally showed lower heterogeneity and more stable performance, underscoring the importance of rigorous validation. Nearly one-third of models were built with EPV <10, a condition known to dramatically increase overfitting risk. Small datasets paired with powerful algorithms (e.g., XGBoost, DNNs) may produce spuriously high AUC values. Notably, subgroup analyses revealed that studies with insufficient EPV contributed disproportionately to heterogeneity.

### 4.4 Methodological quality: persistent issues in prediction modeling

The PROBAST assessment revealed profound methodological weaknesses, with 100% (40/40) of studies judged as having high or unclear risk of bias, predominantly within the analysis domain. This high proportion directly challenges the reliability of the reported predictive performances. Key issues contributing to this bias included inadequate events-per-variable ratios (present in 27.5% of studies), leading to a high risk of overfitting, and poorly addressed or unreported missing data handling (32.5% of development studies). Critically, only 17.5% (7/40) of studies included external validation, and all of these were themselves compromised by small validation cohorts. This pervasive lack of external validation severely limits the generalizability of the models to new patient populations or different healthcare settings, making their real-world performance highly uncertain.

However, beyond these PROBAST-identified issues, our in-depth analysis of ML-specific practices uncovered additional, more granular layers of bias that standard appraisal tools may not fully capture, directly challenging the reliability of the reported high performance. A major concern was the widespread potential for data leakage. Over 60% of studies applied feature selection or data imputation before data splitting. This is a fundamental methodological flaw that violates the principle of independent test sets and almost guarantees overoptimistic results. For instance, a model might appear to have an AUC of 0.96 (Li, 2025) or 0.99 (Lu, 2024), but if feature selection was performed on the entire dataset, these values are likely spurious and would not be replicable in a new population. This practice alone could explain a substantial portion of the high AUCs reported. Furthermore, the use of random cross-validation for time-to-event data, as seen in 80% of relevant studies, is statistically incorrect. This approach fails to account for temporal dependencies and essentially allows models to use information from the future to predict the past, further inflating performance estimates. This is particularly problematic for long-term mortality models (e.g., Guo, 2022, Sun, 2024), where the order of events is critical.

The assessment of reporting quality using the TRIPOD checklist corroborates these methodological concerns, revealing a transparency gap that further hinders reliability and reproducibility. While general study characteristics were well-reported, critical details necessary for model replication or external validation were frequently omitted. For instance, 62.5% of studies failed to provide clear definitions for the predictors used, and 82.5% did not justify their sample size. Most concerning for clinical implementation, 72.5% of studies did not publish the full model specifications (e.g., final coefficients, architecture, hyperparameters), rendering the models “black boxes” that cannot be independently validated or integrated into electronic medical records. Only 30% of studies elaborated on how the model might be used in clinical practice, highlighting a disconnect between model development and clinical applicability.

A critical, and perhaps most consequential, methodological flaw undermining the credibility of the current evidence base is the near-total absence of rigorous external validation. While our PROBAST assessment noted that only 17.5% of studies included any external validation, this statistic does not fully capture the severity of the issue. The majority of these so-called “external validations” were merely temporal splits from the same institution. Models validated in this manner are still vulnerable to institution-specific biases in patient selection, perioperative management, and outcome ascertainment. They provide no evidence that a model will perform reliably in a different hospital, country, or healthcare setting with a different case mix and clinical workflow. Consequently, the generalizability of these models remains entirely unknown. This is particularly problematic for ML models, which are notorious for their ability to fit idiosyncrasies of the training data, a phenomenon often exacerbated by single-center datasets. The few models tested on truly independent, multi-center cohorts (*n* = 2) are the exception that proves the rule, and even these were often hampered by small sample sizes in the validation set, limiting the precision of their performance estimates. Therefore, the field is currently populated by models that are, at best, well-tuned to their local institutional data but of unproven value elsewhere.

A further critical limitation undermining clinical interpretability and utility is the infrequent and incomplete assessment of model calibration and clinical net benefit. Although calibration was assessed in 72.5% of studies, over a quarter (27.5%) relied solely on discrimination metrics, which are insufficient to determine whether a model's predicted probabilities are accurate. A model can have excellent discrimination but poor calibration, which can lead to misguided clinical decisions. Furthermore, decision curve analysis (DCA) was performed in only a small minority of studies (e.g., IDI/NRI reported in only 5% of studies, implying DCA was even rarer). DCA is essential for evaluating the clinical utility of a model by quantifying its net benefit across a range of threshold probabilities. The absence of DCA means that even well-performing models lack evidence that their use would improve clinical decision-making compared to current practice. These omissions collectively prevent a comprehensive understanding of a model's readiness for bedside application, as they fail to demonstrate that the model is both accurate in its risk estimates and beneficial in guiding patient care. Consequently, while the models show promise as research tools, the methodological weaknesses identified mean that the current evidence is insufficient to support their adoption in routine clinical practice.

The handling of class imbalance further compounds these limitations. 14 studies predicted outcomes with low event rates (<20%), such as Jin, 2025 (MMP, 1.6%) and Zhang, 2025 (30-day mortality, 5.8%). Among these, only 5 studies (35.7%) reported using techniques to address imbalance. The remaining studies relied solely on accuracy and AUC, which are known to be misleading in imbalanced datasets. The striking example of Wen, 2025, where the XGBoost model achieved an excellent AUC of 0.969 but a precision of only 49.0% (meaning less than half of the patients flagged as high-risk would actually experience the outcome), perfectly illustrates this point. Such a model, if implemented clinically, could lead to unnecessary interventions and patient anxiety.

Finally, the combination of complex models with small datasets in a quarter of the included studies suggests a high risk of overfitting. For example, studies using deep neural networks (DNN) or complex ensembles on cohorts with fewer than 300 patients (e.g., Chen, 2023, Ma, 2024) are highly susceptible to learning noise rather than true signal. This aligns with our meta-analytic finding of wide prediction intervals, particularly for outcomes like AKI (95% PI: 0.384–0.974), indicating that model performance is highly unstable and context-dependent. These issues, taken together, indicate that the current literature is characterized by a “testing on the training data” mindset, rather than a rigorous focus on generalizability and clinical applicability.

An additional consideration pertains to the source of the included studies. Our search strategy encompassed both peer-reviewed journals and grey literature from CNKI and Wanfang databases, which included Master's theses and dissertations. While the inclusion of such sources enhances the comprehensiveness of the review and mitigates publication bias, it also introduces potential variability in methodological rigor. Non-peer-reviewed studies may not have undergone the same level of critical scrutiny as journal publications, potentially affecting the reliability of their findings.

### 4.5 Clinical implications

Given the high mortality of acute type A AD and the complexity of perioperative management, early identification of high-risk patients is essential. Machine learning models offer a transformative potential in surgical care by stratifying patient surgical urgency, guiding the allocation of critical resources such as ICU-level interventions, predicting perioperative complications to inform targeted preventive strategies, and thereby supporting data-driven, individualized discussions with patients and families regarding procedural risks and expected outcomes. Models integrating preoperative imaging, laboratory data, and hemodynamic features could be particularly valuable in identifying patients at imminent risk of rupture, organ malperfusion, or postoperative complications.

Predictive models for conditions such as acute kidney injury, neurological injury, and multiorgan dysfunction hold significant potential to enhance clinical management by enabling individualized fluid administration, targeted renoprotective strategies, the timely initiation of early mobilization or neuroprotective interventions, and tailored monitoring protocols aligned with projected risk trajectories. Given the high stakes of AD management, the potential of accurate risk prediction is immense. If robustly developed and validated, ML models could indeed transform surgical planning and perioperative care. However, given the current state of the evidence outlined above, these potential benefits remain entirely theoretical. The critical missing link is proven generalizability. A model developed and validated on data from a single high-volume aortic center in Eastern China, for example, cannot be assumed to work in a community hospital in Europe or even in a different tertiary center within the same country. Differences in population genetics, referral patterns, diagnostic pathways, and surgical techniques can all fundamentally alter a model's predictive accuracy. It is crucial for clinicians to understand that no ML-based prediction tool for AD has yet been shown to be consistently reliable across different patient populations and hospital settings. Therefore, these models should not currently be used to guide individual patient decisions. Their role, for now, is confined to the research domain. Premature implementation of these unvalidated models could lead to misguided clinical judgments and potential patient harm.

For real-world adoption, ML models must be embedded into clinical workflows. Future systems could automatically extract relevant variables from electronic medical records (EMRs), run validated algorithms in real time, and display risk predictions on user-friendly dashboards. The successful implementation of such an integrated system necessitates seamless data interoperability, robust real-time computational capabilities, intuitive visualization interfaces, and the capacity for automated recalibration in response to continuously evolving patient population dynamics. Given the complexities of AD management, decision support tools could substantially enhance clinical efficiency and accuracy.

### 4.6 From model to clinic: ethical, explainability, deployment, and regulatory challenges

While the preceding sections have focused on the technical and methodological limitations of current ML models, translating even a perfectly developed model from a research manuscript to the bedside involves overcoming a distinct set of challenges related to ethics, interpretability, deployment, and regulation. The current literature, as reflected in our included studies, has largely neglected these critical translational steps.

Ethical considerations are paramount. The promise of ML models to improve risk stratification must be weighed against the potential for algorithmic bias. Our analysis revealed that the vast majority of studies were conducted in single centers in China, raising concerns about the models' fairness and generalizability to diverse ethnic, socioeconomic, and healthcare populations. A model trained predominantly on data from one demographic group may underperform or even perpetuate existing health disparities when applied to another. Furthermore, the “black box” nature of many ML models creates a fundamental ethical dilemma regarding informed consent and accountability. How can a clinician obtain truly informed consent for a treatment decision influenced by a model whose reasoning is opaque? If a patient suffers an adverse outcome despite the model's prediction, or because of a flawed recommendation, who is accountable—the clinician, the model developer, or the institution that deployed it? These questions of liability and agency remain largely unaddressed in the primary literature we reviewed.

The issue of explainability is central to building clinician trust and enabling safe model deployment. While our review noted the use of SHAP or similar tools in a few recent studies (e.g., Li, 2025, Li, 2025), this remains the exception rather than the rule. For the majority of models (72.5% of which did not publish full specifications), they remain inscrutable “black boxes.” A surgeon is unlikely to alter a high-stakes operative plan based on a risk score from a model that cannot provide a plausible, clinically grounded rationale for its prediction. Explainability is not merely a technical nicety; it is a prerequisite for clinical adoption. It allows clinicians to verify that the model's reasoning aligns with medical knowledge, to identify potential errors, and to communicate risk to patients in a meaningful way. The field must move beyond simply reporting which features are “important” (via SHAP summary plots) to providing faithful, local explanations for individual predictions that clinicians can interrogate and understand.

The obstacles to real-world deployment are formidable and extend far beyond model validation. Our findings show a near-total absence of research on the implementation process. None of the 40 studies, for example, addressed how their model would be integrated into an electronic medical record (EMR) system. In practice, this requires not only technical interoperability but also real-time data extraction, automated preprocessing pipelines, and seamless display of risk predictions within clinical workflows. A model that requires manual data entry or generates results that are not readily accessible at the point of care will never be used. Furthermore, models must be designed to handle the inevitable messiness of real-world data, including variable missingness patterns and data drift over time, a challenge rarely considered in the pristine, research-dataset environment. The successful deployment of an ML tool is as much a socio-technical challenge as it is a technical one, requiring workflow redesign, clinician training, and ongoing monitoring, none of which have been explored in the AD prediction literature.

Finally, the path to clinical use is gated by a complex and evolving regulatory landscape. In most jurisdictions, a ML-based prediction tool intended to guide patient management would be classified as a medical device, requiring rigorous pre-market approval. Regulatory bodies like the U.S. Food and Drug Administration (FDA) are increasingly concerned with the transparency, robustness, and clinical validity of AI-based software. The evidence required for such approval—typically from prospective, multi-center trials demonstrating a meaningful impact on clinical decision-making and patient outcomes—is far beyond what any of the studies in our review have provided. The high risk of bias and poor generalizability we have identified would render these models unsuitable for regulatory submission. Therefore, while the research is promising, it represents a very early stage of a long and arduous journey toward a regulated, deployable clinical tool.

### 4.7 Recommendations for future research

Future studies should ensure an adequate sample size with at least 20 EPV, avoid univariate screening for predictor selection, and appropriately treat continuous predictors by eschewing arbitrary cut-offs. Advanced imputation strategies should be applied to handle missing data, while internal validation ought to rely on bootstrap or repeated cross-validation methods. Additionally, such studies should incorporate calibration metrics and analyses of clinical utility to enhance the robustness and applicability of the findings. Harmonization across studies is essential to ensure robust comparability of clinical findings, which can be achieved by adopting standardized diagnostic criteria for conditions such as acute kidney injury (e.g., KDIGO guidelines), defining uniform time horizons for the assessment of mortality (e.g., 30-day, 1-year, and 3-year endpoints), and publishing consensus-based definitions for composite outcomes. Multicenter datasets would enhance generalizability and help mitigate institutional biases. Federated learning may be a promising approach to enable model development without compromising patient privacy (63). To cultivate clinician trust, future models should publish complete technical specifications, integrate interpretability tools such as SHAP to assess feature importance and clinical plausibility, and provide explicit guidance for clinical application. Ultimately, the value of ML models lies in their ability to improve clinical outcomes. Prospective studies are needed to evaluate whether the model effectively changes clinical decision-making, whether early interventions guided by its machine learning predictions lead to improved patient outcomes, and how the model performs when deployed in real-time clinical settings. Most critically, future research must prioritize rigorous external validation as a non-negotiable step before any model can be considered for clinical use. This requires moving beyond simple temporal validation. Studies should seek to validate models on large, multi-center, and ideally international cohorts that reflect the diversity of real-world clinical practice. Investigators should consider study designs that explicitly test model performance across different subgroups (e.g., by sex, age, dissection type, or treatment modality) and different settings. Furthermore, simply reporting a C-statistic on a validation set is insufficient; studies should assess for calibration drift and the need for model updating or recalibration when applied to new populations. Collaborative efforts and the use of federated learning frameworks could facilitate robust external validation without compromising data privacy. Only through such rigorous testing can we begin to understand which models are truly generalizable and ready for the bedside.

### 4.8 Strengths and limitations of this review

This meta-analysis, the first to comprehensively evaluate machine learning models across multiple AD outcomes, was conducted with rigorous adherence to PRISMA, CHARMS, and TRIPOD guidelines and included a PROBAST assessment of risk of bias, encompassing literature from both English and Chinese sources alongside detailed subgroup and sensitivity analyses. However, several limitations warrant consideration, including heterogeneity in clinical settings and outcome definitions, detected publication bias for acute kidney injury outcomes, a constrained ability to assess model calibration across all included studies, and the possibility that performance estimates may be inflated due to methodological weaknesses in the primary studies. The inclusion of grey literature (e.g., Master's theses from CNKI and Wanfang) may introduce variability in study quality. Specifically, the significant publication bias detected for the AKI outcome suggests that the pooled estimate for AKI should be interpreted with caution, as it is likely inflated by the preferential publication of studies with favorable results. A formal bias-adjusted sensitivity analysis, while desirable, was not feasible due to the limited number of studies available for this outcome. Importantly, our ability to draw conclusions about the generalizability of these ML models is severely limited by the lack of robust external validation in the original studies. The pooled estimates we provide largely reflect internally validated performance and are likely optimistic proxies for real-world effectiveness.

## 5 Conclusion

In summary, this review demonstrates that machine learning-based models show considerable promise for research and hypothesis generation in predicting adverse outcomes for patients with aortic dissection. The consistently high reported AUCs suggest that these techniques are capable of capturing complex signals in clinical data.

However, confidence in this promising evidence base is severely constrained by pervasive methodological flaws and poor reporting transparency. Our analysis revealed that 100% of studies exhibit high or unclear risk of bias (primarily in the PROBAST analysis domain), and over 60% contain critical omissions regarding predictor definitions and model specifications according to TRIPOD guidelines. This promise is further tempered by substantial methodological weaknesses, including the lack of external validation in over 80% of studies and the frequent omission of calibration and decision-curve analyses. These deficiencies collectively undermine the reliability, generalizability, and clinical interpretability of the models, raising significant concerns about the validity of their reported high performance.

Consequently, we conclude that current evidence does not support the routine clinical implementation of any ML-based prediction model for aortic dissection. To bridge the gap between promising research and trustworthy clinical tools, future research must move beyond simply reporting high internal performance. Instead, it must prioritize rigorous analytic methods, transparent reporting, and—most critically—multicenter external validation that demonstrates a tangible impact on clinical decision-making and patient outcomes before these tools can be translated from the research setting to the bedside.

## Data Availability

The original contributions presented in the study are included in the article/Supplementary Material, further inquiries can be directed to the corresponding author.
