# Supplementary material for: Machine learning approaches for risk prediction in aortic dissection: a systematic review and meta-analysis
Source: Front Cardiovasc Med. 2026 Mar 26;13:1777734. doi: 10.3389/fcvm.2026.1777734 (PMC13062221; doi:10.3389/fcvm.2026.1777734)
Supplement: Supplementary file 1 [file Supplementaryfile1.docx]

**Supplementary File 1: Justification of exclusions at full-text review**

**Ineligible study outcome** **(n=1)**

1. Chang H. Y. Stent Selection for Zone 2 Thoracic Endovascular Aortic Repair and Construction of a Machine Learning Model for Predicting Bird-Beak Configuration Risk[D]. Shandong University, 2022.

**Ineligible subject** **(n=1)**

1. Song J. Biomechanical Study on Reintervention After Stanford Type A Aortic Dissection Surgery and Predictive Model for Thoracoabdominal Aortic Replacement Outcomes[D]. Peking Union Medical College, 2023.

**No predictors** **(n=1)**

1. Zhou M, Luo X, Wang X, Xie T, Wang Y, Shi Z, Wang M, Fu W. Deep Learning Prediction for Distal Aortic Remodeling After Thoracic Endovascular Aortic Repair in Stanford Type B Aortic Dissection. J Endovasc Ther. 2024 Oct;31(5):910-918. doi: 10.1177/15266028231160101. Epub 2023 Mar 16. PMID: 36927177.

**Non-machine learning** **(n=10)**

1. Liu G. Z. Analysis of perioperative risk factors and construction of a prediction model for acute kidney injury after acute Stanford A type aortic dissection surgery[D]. Lanzhou University, 2024.
2. Lan T. Analysis of Perioperative Risk Factors and Construction of a Prediction Model for 30-Day Mortality after Acute Type A Aortic Dissection Surgery[D]. Kunming University of Science and Technology, 2024.
3. Chen Y. Analysis of perioperative risk factors and construction of a prediction model for 30-day mortality after acute Stanford type A aortic dissection surgery [D]. Lanzhou University; 2023. DOI: 10.27204/d.cnki.glzhu.2023.001521.
4. Wang MM, Gai MT, Wang BZ, Yesitayi G, Ma YT, Ma X. A prediction model to predict in-hospital mortality in patients with acute type B aortic dissection. BMC Cardiovasc Disord. 2023 May 17;23(1):257. doi: 10.1186/s12872-023-03260-5. PMID: 37198546; PMCID: PMC10193648.
5. Liu H, Qian SC, Zhang YY, Wu Y, Hong L, Yang JN, Zhong JS, Wang YQ, Wu DK, Fan GL, Chen JQ, Zhang SQ, Peng XX, Shao YF, Li HY, Zhang HJ. A Novel Inflammation-Based Risk Score Predicts Mortality in Acute Type A Aortic Dissection Surgery: The Additive Anti-inflammatory Action for Aortopathy and Arteriopathy Score. Mayo Clin Proc Innov Qual Outcomes. 2022 Sep 25;6(6):497-510. doi: 10.1016/j.mayocpiqo.2022.08.005. Erratum in: Mayo Clin Proc Innov Qual Outcomes. 2024 Sep 16;8(5):481. doi: 10.1016/j.mayocpiqo.2024.08.002. PMID: 36185465; PMCID: PMC9519496.
6. Lin H, Chang Y, Guo H, Qian X, Sun X, Yu C. Prediction Nomogram for Postoperative 30-Day Mortality in Acute Type A Aortic Dissection Patients Receiving Total Aortic Arch Replacement With Frozen Elephant Trunk Technique. Front Cardiovasc Med. 2022 Jun 10;9:905908. doi: 10.3389/fcvm.2022.905908. PMID: 35757328; PMCID: PMC9226415.
7. Jiao R, Liu M, Lu X, Zhu J, Sun L, Liu N. Development and Validation of a Prognostic Model to Predict the Risk of In-hospital Death in Patients With Acute Kidney Injury Undergoing Continuous Renal Replacement Therapy After Acute Type a Aortic Dissection. Front Cardiovasc Med. 2022 May 2;9:891038. doi: 10.3389/fcvm.2022.891038. PMID: 35586649; PMCID: PMC9108198.
8. Diao YF, Chen ZB, Gu JX, Xu XY, Lin WF, Yuan CZ, Xiong JQ, Li MH, Ni BQ, Zhao S, Shao YF, Zhang YY, Liu H. Incorporating Circulating Plasma Interleukin-10 Enhanced Risk Predictability of Mortality in Acute Type A Aortic Dissection Surgery. Rev Cardiovasc Med. 2025 Feb 21;26(2):26334. doi: 10.31083/RCM26334. PMID: 40026520; PMCID: PMC11868896.
9. Cai H, Shao Y, Li ZH, Liu XY, Zhao XZ, Li CY, Ran HY, Zhou RQ, Shi HM, Shuangling S, Duan CZ, Wu QC, Zhang C. Prognostic prediction of long-term survival in patients with type A aortic dissection undergoing surgical repair: development of a novel prognostic index. BMC Cardiovasc Disord. 2025 Feb 13;25(1):99. doi: 10.1186/s12872-025-04552-8. PMID: 39948452; PMCID: PMC11823025.
10. Jiao R, Liu M, Lu X, Zhu J, Sun L, Liu N. A nomogram for reduced cardiac function in postoperative acute type A aortic dissection patients with acute kidney injury undergoing continuous renal replacement therapy. Front Cardiovasc Med. 2022 Jul 22;9:874715. doi: 10.3389/fcvm.2022.874715. PMID: 35942182; PMCID: PMC9356236.
