# Supplementary material for: Machine learning approaches for risk prediction in aortic dissection: a systematic review and meta-analysis
Source: Front Cardiovasc Med. 2026 Mar 26;13:1777734. doi: 10.3389/fcvm.2026.1777734 (PMC13062221; doi:10.3389/fcvm.2026.1777734)
Supplement: Supplementary file 3 [file Table2.docx]

**Supplementary Table S2. CHARMS 2014 Checklist**

| **Domain** | **Key Items** | **Reported on Page No.** |
| --- | --- | --- |
| **Source of data** | Source of data (e.g., cohort, case-control, randomized trial participants, or registry data) | Methods |
| **Participants** | Participant eligibility and recruitment method (e.g., consecutive participants, location, number of centers, setting, inclusion and exclusion criteria) | Methods |
|  | Participant description | Results |
|  | Details of treatments received, if relevant | Not Applicable |
|  | Study dates | Methods |
| **Outcome(s) to be predicted** | Definition and method for measurement of outcome | Methods |
|  | Was the same outcome definition (and method for measurement) used in all patients? | Methods |
|  | Type of outcome (e.g., single or combined endpoints) | Methods |
|  | Was the outcome assessed without knowledge of the candidate predictors (i.e., blinded)? | Methods |
|  | Were candidate predictors part of the outcome (e.g., in panel or consensus diagnosis)? | Methods |
|  | Time of outcome occurrence or summary of duration of follow-up | Methods |
| **Candidate predictors (or index tests)** | Number and type of predictors (e.g., demographics, patient history, physical examination, additional testing, disease characteristics) | Results |
|  | Definition and method for measurement of candidate predictors | Results |
|  | Timing of predictor measurement (e.g., at patient presentation, at diagnosis, at treatment initiation) | Results |
|  | Were predictors assessed blinded for outcome, and for each other (if relevant)? | Results |
|  | Handling of predictors in the modeling (e.g., continuous, linear, nonlinear transformations, or categorized) | Results |
| **Sample size** | Number of participants and number of outcomes/events | Results |
|  | Number of outcomes/events in relation to the number of candidate predictors (events per variable) | Table 2 |
| **Missing data** | Number of participants with any missing value (include predictors and outcomes) | Results |
|  | Number of participants with missing data for each predictor | Results |
|  | Handling of missing data (eg, complete-case analysis, imputation, or other methods) | Results |
| **Model development** | Modelling method (e.g., logistic, survival, neural network, or machine learning techniques) | Results |
|  | Modelling assumptions satisfied | Results |
|  | Method for selection of predictors for inclusion in multivariable modelling (e.g., all candidate predictors, pre-selection based on unadjusted association with the outcome) | Results |
|  | Method for selection of predictors during multivariable modeling (e.g., full model approach, backward or forward selection) and criteria used (e.g., p-value, Akaike information criterion) | Results |
|  | Shrinkage of predictor weights or regression coefficients (e.g., no shrinkage, uniform shrinkage, penalized estimation) | Results |
| **Model performance** | Calibration (calibration plot, calibration slope, Hosmer-Lemeshow test) and Discrimination (C-statistic, D-statistic, log-rank) measures with confidence intervals | Results |
|  | Classification measures (e.g., sensitivity, specificity, predictive values, net reclassification improvement) and whether a-priori cut points were used | Results |
| **Results** | Final and other multivariable models (e.g., basic, extended, simplified) presented, including predictor weights or regression coefficients, intercept, baseline survival, model performance measures (with standard errors or confidence intervals) | Table 3 |
|  | Any alternative presentation of the final prediction models, e.g., sum score, nomogram, score chart, predictions for specific risk subgroups with performance NA | Table 3 |
|  | Comparison of the distribution of predictors (including missing data) for development and validation datasets | Not Applicable |
| **Interpretation and discussion** | Interpretation of presented models (confirmatory, i.e., model useful for practice versus exploratory, i.e., more research needed) | Discussion |
|  | Comparison with other studies, discussion of generalizability, strengths and limitations | Discussion |
