# Supplementary material for: Machine learning approaches for risk prediction in aortic dissection: a systematic review and meta-analysis
Source: Front Cardiovasc Med. 2026 Mar 26;13:1777734. doi: 10.3389/fcvm.2026.1777734 (PMC13062221; doi:10.3389/fcvm.2026.1777734)
Supplement: Supplementary file 5 [file Table4.docx]

**Supplementary Table S4.** **Complete list of search terms**

| Search Terms |
| --- |
| (“machine learning”[Title/Abstract] OR “artificial intelligence”[Title/Abstract] OR “deep learning”[Title/Abstract] OR “neural network*”[Title/Abstract])  AND  (“risk prediction”[Title/Abstract] OR “prognosis”[Title/Abstract] OR “mortality”[Title/Abstract] OR “complication*”[Title/Abstract] OR “adverse event*”[Title/Abstract] OR “readmission”[Title/Abstract])  AND  ("aortic dissection"[MeSH Terms] OR "aortic dissection"[Title/Abstract] OR "Stanford type A"[Title/Abstract] OR "Stanford type B"[Title/Abstract] OR TEVAR[Title/Abstract])  NOT (animals[MeSH] NOT humans[MeSH]) |

MESH, Medical Subject Headings

| **Number** | **Search Terms** | **Medline (PubMed)** | **Embase** | **Web of Science** | **Cochrane** | **CNKI** | **Wanfang** |
| --- | --- | --- | --- | --- | --- | --- | --- |
| 1 | ((machine learning[Title/Abstract]) OR (artificial intelligence[Title/Abstract]) OR (deep learning[Title/Abstract]) OR (neural network*[Title/Abstract])) | 540,830 | 312,775 | 427,619 | 10,751 | 945,213 | 113,255 |
| 2 | ((risk prediction[Title/Abstract] OR (prognosis[Title/Abstract]) OR mortality[Title/Abstract] OR (complication*[Title/Abstract]) OR (adverse event*[Title/Abstract]) OR (readmission[Title/Abstract])) | 7,020,118 | 495,123 | 734,219 | 545,417 | 2,460,981 | 4,299,886 |
| 3 | ((aortic dissection[MeSH Terms] OR aortic dissection[Title/Abstract] OR Stanford type A[Title/Abstract] OR Stanford type B[Title/Abstract] OR TEVAR[Title/Abstract])) | 68,610 | 31,582 | 18,637 | 1,136 | 16,010 | 24,359 |
| 4 | 1 AND 2 AND 3 | 372 | 28 | 53 | 5 | 37 | 36 |

Total = 531

After removal of duplicates (Endnote) = 507

Full text review = 40
