# Supplementary material for: Machine learning approaches for risk prediction in aortic dissection: a systematic review and meta-analysis
Source: Front Cardiovasc Med. 2026 Mar 26;13:1777734. doi: 10.3389/fcvm.2026.1777734 (PMC13062221; doi:10.3389/fcvm.2026.1777734)
Supplement: Supplementary file 8 [file Table7.docx]

**Supplementary Table S7. Subgroup analysis of machine learning models for early mortality prediction in aortic dissection patients**

| **Category** | **Subgroups** | **No studies** | **Heterogeneity test** | | **Meta-analysis** |
| --- | --- | --- | --- | --- | --- |
|  |  |  | ***I^2^（%）*** | ***P*** | ***OR（95%CI）*** |
| Participants population | AAD | 5 | 73.5 | 0.005 | 0.87 (0.79, 0.92) |
|  | ATAAD | 3 | 0 | 0.968 | 0.92 (0.89, 0.94) |
|  | AD | 1 | Not applicable | | |
| Sample size | ≥1000 | 3 | 100 | ＜0.001 | 0.89 (0.82, 0.93) |
|  | ＜1000 | 6 | 71.2 | 0.004 | 0.89 (0.80, 0.94) |
| EPV | ＜10 | 3 | 100 | ＜0.001 | 1.00 (0.82, 0.96) |
|  | 10~20 | 4 | 100 | ＜0.001 | 0.90 (0.83, 0.94) |
|  | ＞20 | 2 | 97.2 | ＜0.001 | 0.85 (0.83, 0.86) |
| Model development method | LR only | 1 | Not applicable | | |
|  | LR + ML models | 7 | 77.6 | ＜0.001 | 0.89 (0.84, 0.95) |
|  | ML models only | 1 | Not applicable | | |
| Validation approach | Hold-out | 4 | 77.3 | 0.032 | 0.86 (0.78, 0.94) |
|  | Cross-validation | 2 | 72.5 | 0.057 | 0.89 (0.80, 0.97) |
|  | Hold-out + External validation | 2 | 0 | 0.458 | 0.92 (0.89, 0.94) |
|  | Validation not reported | 1 | Not applicable | | |
| **Abbreviations:** AAD: acute aortic dissection; AD: aortic dissection; ATAAD, acute type A aortic dissection; CI: confidence interval; I²: I-squared; LR: logistic regression; ML: machine learning; No.: number; OR: odds ratio | | | | | |
