# Supplementary material for: Machine learning approaches for risk prediction in aortic dissection: a systematic review and meta-analysis
Source: Front Cardiovasc Med. 2026 Mar 26;13:1777734. doi: 10.3389/fcvm.2026.1777734 (PMC13062221; doi:10.3389/fcvm.2026.1777734)
Supplement: Supplementary file 9 [file Table8.docx]

**Supplementary Table S8. Subgroup analysis of machine learning models for long-term mortality prediction in aortic dissection patients**

| **Category** | **Subgroups** | **No studies** | **Heterogeneity test** | | **Meta-analysis** |
| --- | --- | --- | --- | --- | --- |
|  |  |  | ***I^2^（%）*** | ***P*** | ***OR（95%CI）*** |
| Participants population | TAAD | 2 | 70.4 | 0.0661 | 0.87 (0.82, 0.93) |
|  | AAD | 2 | 40.8 | 0.1940 | 0.86 (0.77, 0.91) |
|  | ATAAD | 1 | Not applicable | | |
| Sample size | ≥500 | 2 | 81.1 | 0.0220 | 0.83 (0.64, 0.93) |
|  | ＜500 | 3 | 0 | 0.2326 | 0.85 (0.85, 0.86) |
| EPV | ＜10 | 2 | 0 | 0.3904 | 0.85 (0.85, 0.86) |
|  | 10~20 | 2 | 0 | 0.8223 | 0.91 (0.85, 0.94) |
|  | NI | 1 | Not applicable | | |
| Validation approach | Hold-out | 2 | 0 | 1.0000 | 0.83 (0.76, 0.89) |
|  | Cross-validation | 2 | 0 | 0.8237 | 0.91 (0.86, 0.95) |
|  | External validation | 1 | Not applicable | | |
| **Abbreviations:** AAD: acute aortic dissection; ATAAD, acute type A aortic dissection; CI: confidence interval; I²: I-squared; NI: not information; No.: number; OR: odds ratio; TAAD: type A aortic dissection | | | | | |
