# Supplementary material for: Machine learning approaches for risk prediction in aortic dissection: a systematic review and meta-analysis
Source: Front Cardiovasc Med. 2026 Mar 26;13:1777734. doi: 10.3389/fcvm.2026.1777734 (PMC13062221; doi:10.3389/fcvm.2026.1777734)
Supplement: Supplementary file 10 [file Table9.docx]

**Supplementary Table S9. Subgroup analysis of machine learning models for AKI prediction in aortic dissection patients**

| **Category** | **Subgroups** | **No studies** | **Heterogeneity test** | | **Meta-analysis** |
| --- | --- | --- | --- | --- | --- |
|  |  |  | ***I^2^（%）*** | ***P*** | ***OR（95%CI）*** |
| Participants population | AAD | 2 | 0 | 0.671 | 0.74 (0.65, 0.81) |
|  | ATAAD | 3 | 76.8 | 0.02 | 0.88 (0.81, 0.93) |
| Sample size | ≥1000 | 1 | Not applicable | | |
|  | 500~1000 | 1 | Not applicable | | |
|  | ＜500 | 3 | 0 | 0.662 | 0.76 (0.68, 0.82) |
| EPV | ＜10 | 1 | Not applicable | | |
|  | 10~20 | 2 | 90.5 | 0.001 | 0.86 (0.65, 0.95) |
|  | ＞20 | 2 | 40.7 | 0.194 | 0.85 (0.78, 0.91) |
| Validation approach | Cross-validation | 3 | 67.8 | 0.035 | 0.81 (0.72, 0.90) |
|  | Bootstrap | 1 | Not applicable | | |
|  | Mixed（CV + Bootstrap） | 1 | Not applicable | | |
| Abbreviations: AAD: acute aortic dissection; ATAAD, acute type A aortic dissection; CI: confidence interval; CR: cardiac rupture; FWR: free-wall rupture; I²: I-squared; No.: number; OR: odds ratio; STEMI: ST-elevation myocardial infarction | | | | | |
