# Supplementary material for: Machine learning approaches for risk prediction in aortic dissection: a systematic review and meta-analysis
Source: Front Cardiovasc Med. 2026 Mar 26;13:1777734. doi: 10.3389/fcvm.2026.1777734 (PMC13062221; doi:10.3389/fcvm.2026.1777734)
Supplement: Supplementary file 11 [file Table10.docx]

**Supplementary Table S10.** **Risk of bias assessment tables and key for risk of bias assessment**

**Risk of bias assessment: population, predictors and outcomes (based on PROBAST)**

| **Study** | **Model** | | **Appropriate data source?** | **In/exclusions of participants appropriate?** | **Predictors defined and assessed in similar way for all participants?** | **Predictor assessment made without knowledge of outcome data?** | **All predictors available at the time the model is intended to be used?** | **Outcome determined appropriately?** | **Pre-defined/standard outcome definition?** | **Predictors excluded from the outcome definition?** | **Outcome defined and determined in a similar way for all participants?** | **Outcome determined without knowledge of predictor information?** | **Appropriate time interval between predictor and outcome assessment?** |
| --- | --- | --- | --- | --- | --- | --- | --- | --- | --- | --- | --- | --- | --- |
| Cai H *et al.* |  | **DEV √**  **INT VAL √**  **EXT VAL √**  U/M X  COMP X | PY | PY | PY | Y | Y | Y | PY | Y | PY | Y | Y |
| Chen H *et al.* |  | **DEV √**  **INT VAL √**  EXT VAL X  U/M X  COMP X | PY | PY | PY | PY | Y | Y | PY | Y | PY | NI | PY |
| Chen Q *et al.* |  | **DEV √**  **INT VAL √**  EXT VAL X  U/M X  COMP X | PY | PY | PY | PY | Y | PY | PY | Y | PY | Y | Y |
| Chen Q *et al.* |  | **DEV √**  **INT VAL √**  EXT VAL X  U/M X  COMP X | PY | Y | Y | Y | Y | Y | Y | Y | PY | Y | Y |
| Chen Z *et al.* |  | **DEV √**  **INT VAL √**  EXT VAL X  U/M X  COMP X | PY | PY | PY | PY | Y | PY | PY | Y | PY | NI | Y |
| Dai A *et al.* |  | **DEV √**  **INT VAL √**  EXT VAL X  U/M X  COMP X | PY | PY | Y | PY | Y | Y | PY | Y | Y | NI | Y |
| Dong Y *et al.* |  | **DEV √**  **INT VAL √**  EXT VAL X  U/M X  COMP X | PY | PY | PY | PY | Y | PY | PY | Y | PY | NI | Y |
| Guo T *et al.* |  | **DEV √**  **INT VAL √**  EXT VAL X  U/M X  COMP X | PY | PY | PY | PY | Y | PY | Y | Y | PY | Y | Y |
| Guo Z *et al.* |  | **DEV √**  **INT VAL √**  EXT VAL X  U/M X  COMP X | Y | PY | Y | Y | Y | PY | Y | Y | PY | Y | Y |
| He X *et al.* |  | **DEV √**  **INT VAL √**  EXT VAL X  U/M X  COMP X | Y | PY | PY | PY | Y | PY | Y | Y | PY | PY | Y |
| Jiang Y *et al.* |  | **DEV √**  **INT VAL √**  EXT VAL X  U/M X  COMP X | PY | PY | PY | Y | Y | PY | Y | Y | PY | Y | Y |
| Jin Z *et al.* |  | **DEV √**  **INT VAL √**  EXT VAL X  U/M X  COMP X | PY | PY | PY | PY | Y | PY | Y | Y | PY | NI | PY |
| Jin Z *et al.* |  | **DEV √**  **INT VAL √**  **EXT VAL √**  U/M X  COMP X | PY | PY | PY | PY | Y | PY | PY | Y | PY | NI | PY |
| Lei J *et al.* |  | **DEV √**  **INT VAL √**  **EXT VAL √**  U/M X  COMP X | PY | PY | PY | Y | Y | PY | Y | Y | PY | Y | Y |
| Li K *et al.* |  | **DEV √**  **INT VAL √**  EXT VAL X  U/M X  COMP X | PY | PY | PY | PY | Y | PY | PY | Y | PY | NI | PY |
| Li L *et al.* |  | **DEV √**  **INT VAL √**  EXT VAL X  U/M X  COMP X | PY | PY | PY | PY | Y | PY | Y | Y | PY | NI | Y |
| Li L *et al.* |  | **DEV √**  **INT VAL √**  EXT VAL X  U/M X  COMP X | PY | PY | PY | PY | Y | PY | PY | Y | PY | NI | PY |
| Li P *et al.* |  | **DEV √**  INT VAL X  EXT VAL X  U/M X  COMP X | PY | PY | PY | PY | Y | PY | Y | Y | PY | Y | Y |
| Li X *et al.* |  | **DEV √**  **INT VAL √**  EXT VAL X  U/M X  COMP X | PY | PY | PY | PY | Y | PY | PY | Y | PY | NI | Y |
| Lin Y *et al.* |  | **DEV √**  **INT VAL √**  EXT VAL X  U/M X  COMP X | PY | PY | PY | PY | Y | PY | PY | Y | PY | PY | PY |
| Liu X *et al.* |  | **DEV √**  **INT VAL √**  EXT VAL X  U/M X  COMP X | PY | PY | PY | PY | Y | PY | PY | Y | PY | NI | PY |
| Lu X *et al.* |  | **DEV √**  **INT VAL √**  **EXT VAL √**  U/M X  COMP X | PY | PY | PY | PY | Y | PY | PY | Y | PY | NI | PY |
| Luo H *et al.* |  | **DEV √**  **INT VAL √**  EXT VAL X  U/M X  COMP X | PY | PY | PY | PY | Y | PY | PY | Y | PY | NI | PY |
| Ma Y *et al.* |  | **DEV √**  INT VAL X  EXT VAL X  U/M X  COMP X | PY | PY | PY | PY | Y | PY | PY | Y | PY | NI | PY |
| Pang L *et al.* |  | **DEV √**  **INT VAL √**  EXT VAL X  U/M X  COMP X | PY | PY | PY | PY | Y | PY | PY | Y | PY | PY | PY |
| Pei W *et al.* |  | **DEV √**  **INT VAL √**  EXT VAL X  U/M X  COMP X | PY | PY | PY | PY | Y | PY | Y | Y | PY | Y | Y |
| Song X *et al.* |  | **DEV √**  **INT VAL √**  EXT VAL X  U/M X  COMP X | PY | PY | PY | PY | Y | PY | Y | Y | PY | Y | Y |
| Sun Q *et al.* |  | **DEV √**  **INT VAL √**  EXT VAL X  U/M X  COMP X | PY | PY | PY | PY | Y | PY | Y | Y | PY | Y | Y |
| Wang M *et al.* |  | **DEV √**  **INT VAL** X  EXT VAL X  U/M X  COMP X | PY | PY | PY | PY | Y | Y | Y | Y | PY | NI | Y |
| Wang Y *et al.* |  | **DEV √**  **INT VAL** X  EXT VAL X  U/M X  COMP X | PY | PY | PY | PY | Y | PY | PY | Y | PY | Y | Y |
| Wei Z *et al.* |  | **DEV √**  **INT VAL √**  **EXT VAL √**  U/M X  COMP X | PY | PY | PY | PY | Y | PY | Y | Y | PY | NI | Y |
| Wei Z *et al.* |  | **DEV √**  **INT VAL √**  EXT VAL X  U/M X  COMP X | PY | PY | PY | PY | Y | PY | PY | Y | PY | NI | PY |
| Wen S *et al.* |  | **DEV √**  **INT VAL √**  **EXT VAL √**  U/M X  COMP X | PY | PY | PY | PY | Y | PY | PY | Y | PY | NI | PY |
| Wu Z *et al.* |  | **DEV √**  **INT VAL √**  EXT VAL X  U/M X  COMP X | PY | PY | PY | PY | Y | PY | Y | Y | PY | Y | Y |
| Xie L *et al.* |  | **DEV √**  **INT VAL √**  EXT VAL X  U/M X  COMP X | PY | PY | PY | PY | Y | PY | Y | Y | PY | Y | Y |
| Xie L *et al.* |  | **DEV √**  **INT VAL √**  EXT VAL X  U/M X  COMP X | PY | PY | PY | PY | Y | PY | Y | Y | PY | NI | Y |
| Zhang J *et al.* |  | **DEV √**  **INT VAL √**  EXT VAL X  U/M X  COMP X | PY | PN | PY | PY | Y | PY | Y | Y | PY | Y | Y |
| Zhang S *et al.* |  | **DEV √**  **INT VAL √**  EXT VAL X  U/M X  COMP X | PY | PY | PY | PY | Y | PY | Y | Y | PY | Y | Y |
| Zhang X *et al.* |  | **DEV √**  **INT VAL √**  **EXT VAL √**  U/M X  COMP X | PY | PY | Y | Y | Y | PY | Y | Y | PY | Y | Y |
| Zhao H *et al.* |  | **DEV √**  **INT VAL √**  EXT VAL X  U/M X  COMP X | PY | PY | PY | PY | Y | PY | PY | Y | PY | NI | Y |

AF = atrial fibrillation; CABG = coronary artery bypass graft; CAD = coronary artery disease; COMP=study which compares two or more models; DEV=model development study; EDW = Enterprise Data Warehouse; EXT VAL =study with external validation of a model; INT VAL= study with internal model validation; LVEF=left ventricular ejection fraction; N=no; NEI=not enough information; NI=no information; PCI = Percutaneous Coronary Intervention; PN=probably no; PY=probably yes; U/M =study which updates or modifies a model; Y=yes.

* relates to postoperative stroke, postoperative potassium, postoperative low cardiac output, length of hospital stay, postoperative IABP assistance, ICU stay time, ventilator support time, postoperative β-blocker treatment, postoperative ACEI treatment, postoperative potassium supplementation, postoperative NSAIDs treatment, as a variable for prediction of POAF, so variables cannot be used pre-procedurally

**Risk of bias assessment: analysis (based on PROBAST)**

| **Study** | **Model** | **Was there a reasonable number of participants with the outcome?** | **Were continuous and categorical predictors handled appropriately? For validation: was model evaluated as originally fitted?** | **Were all enrolled participants included in the analysis?** | **Were participants with missing data handled appropriately?** | **Was selection of predictors based on univariate analysis avoided? (DEV only)** | **Were complexities in the data (e.g. censoring, competing risks, sampling of control participants) accounted for appropriately?*** | **Were relevant model performance measures evaluated appropriately?** | **Were model overfitting and optimism in model performance accounted for? (DEV only)** | **Do predictors and their assigned weights in the final model correspond to the results from the reported multivariable analysis? (DEV only)** | **Where applicable: Appropriate quantification of added value (0ne score compared to another)? Appropriate method of updating model?** |
| --- | --- | --- | --- | --- | --- | --- | --- | --- | --- | --- | --- |
| Cai H *et al.*  DEV | **DEV √**  **INT VAL √**  **EXT VAL √**  U/M X  COMP X | N  7 variables; 53 events. 8 EPV. | Y | NI  27/300 (9.0%) excluded for incomplete record | Y  Random forest algorithms was applied to variables with <20% missing data, whereas those with a proportion exceeding 20% were excluded. | Y  LASSO Cox regression analysis, univariate analysis and correlation analysis | NI | Y | Y  Hold-out validation, 10-fold cross-validation | NI | N/A |
| Cai H *et al.*  EXT VAL |  | N for EXT VAL  73 sample size | Y |  |  |  | N/A  No model refitting |  |  |  |  |
| Chen H *et al.* | **DEV √**  **INT VAL** X  EXT VAL X  U/M X  COMP X | N  7 variables; 27 events. 4 EPV. | Y | NI | N | Y  LASSO to identify variables | NI | Y | PN  Hold-out validation | NI | N/A |
| Chen Q *et al.* | **DEV √**  **INT VAL** X  EXT VAL X  U/M X  COMP X | NI  12 variables. | Y | NI | Y  Features with missing values more than 20% were excluded. Missing data were assumed to be missing at random and were imputed using 10-fold multiple imputation by chained equations. | Y  Kendall correlation coefficient to identify variables | NI | N  No calibration measures reported. | Y  5-fold cross-validation | NI | N/A |
| Chen Q *et al.* | **DEV √**  **INT VAL** X  EXT VAL X  U/M X  COMP X | PY  8 variables; 84 events. 11 EPV. | Y | Y | Y  MissForest imputation method was applied to variables with <10% missing data, whereas those with a proportion exceeding 10% were excluded. | Y  Univariate and multivariate binary logistic regression analyses, DT, RF, XGBoost, SVM to identify variables | NI | Y | N | Y | N/A |
| Chen Z *et al.* | **DEV √**  **INT VAL** X  EXT VAL X  U/M X  COMP X | PY  10 variables; 586 events. 59 EPV. | Y | NI  1/1535 (0.001%) excluded for incomplete record | Y  Variables with more than 20% missing data were excluded from further analysis. For variables with missing data below this threshold, multiple imputation was employed to minimize bias. | NI | NI | Y | Y  10-fold cross-validation | NI | N/A |
| Dai A *et al.* | **DEV √**  **INT VAL** X  EXT VAL X  U/M X  COMP X | Y  9 variables; 191 events. 21 EPV. | Y | NI  8/306 (2.6%) excluded for incomplete record | Y  KNN method was applied to fill in the missing data. | Y  LR, LASSO to identify variables | NI | Y | Y  10-fold cross-validation | NI | N/A |
| Dong Y *et al.* | **DEV √**  **INT VAL** X  EXT VAL X  U/M X  COMP X | PY  7 variables; 68 events. 10 EPV. | Y | N  86/312 (27.6%) excluded for incomplete record | N  Excluded | Y  LASSO regression analysis to identify variables | NI | Y | Y  5-fold cross-validation and bootstrapping | Y | N/A |
| Guo T *et al.* | **DEV √**  **INT VAL** X  EXT VAL X  U/M X  COMP X | PY  20 variables; 273 events. 14 EPV. | Y | NI | Y  Imputed values (which were combined using Rubin’s rules) were used to impute variables with <10% missing data, whereas those with a proportion exceeding 10% were excluded. | NI | NI | N  No calibration measures reported. | Y  10-fold cross-validation | NI | N/A |
| Guo Z *et al.* | **DEV √**  **INT VAL** X  EXT VAL X  U/M X  COMP X | PY  9 variables; 142 events. 16 EPV. | Y | NI | Y  Multiple imputation was applied to variables with <20% missing data, whereas those with a proportion exceeding 20% were excluded. | Y  LASSO regression analysis to identify variables | NI | Y | Y  10-fold cross-validation | PY | N/A |
| He X *et al.* | **DEV √**  **INT VAL** X  EXT VAL X  U/M X  COMP X | N  12 variables; 44 events. 4 EPV. | Y | NI | Y  Multiple imputation was applied to variables with <10% missing data, whereas those with a proportion exceeding 10% were excluded. | Y  CoxBoost, StepCox, RSF, LASSO to identify variables | NI | Y | N  Hold-out validation | PY | N/A |
| Jiang Y *et al.* | **DEV √**  **INT VAL** X  EXT VAL X  U/M X  COMP X | Y  9 variables; 205 events. 23 EPV. | Y | NI | Y  Multiple imputation was applied to variables with <10% missing data, whereas those with a proportion exceeding 20% were excluded. | Y  Backward stepwise regression, LASSO,  BSS, RF, AdaBoost, Weighted k-NN, SVM, NN to identify variables | NI | N  No calibration measures reported. | Y  5-fold cross-validation | NI | N/A |
| Jin Z *et al.* | **DEV √**  **INT VAL** X  EXT VAL X  U/M X  COMP X | Y  5 variables; 87 events. 17 EPV. | Y | NI | Y  We addressed missing values through multiple imputation. | Y  LASSO to identify variables | NI | Y | Y  5-fold cross-validation | PY | N/A |
| Jin Z *et al.*  DEV | **DEV √**  **INT VAL √**  **EXT VAL √**  U/M X  COMP X | Y  4 variables; 105 events. 26 EPV. | Y | NI | Y  Variables with >10% missing data were excluded before further analysis. We subsequently addressed missing values in clinical variables identified during the model-building process using multiple imputation | Y  Univariate and multivariate analyses | NI | Y | Y  5-fold cross-validation and external temporal validation | NI | N/A |
| Jin Z *et al.*  EXT VAL |  | N for EXT VAL  75 sample size | Y |  |  |  | N/A  No model refitting |  |  |  |  |
| Lei J *et al.*  DEV | **DEV √**  **INT VAL √**  **EXT VAL √**  U/M X  COMP X | N  26 variables; 152 events. 6 EPV. | Y | NI | NI | N  Variables chosen on basis of univariate analysis. | NI | N  No calibration measures reported. | N  Hold-out validation; 2 EPV | NI | N/A |
| Lei J *et al.*  EXT VAL |  | N for EXT VAL  188 sample size | Y |  |  |  | N/A  No model refitting |  |  |  |  |
| Li K *et al.* | **DEV √**  **INT VAL** X  EXT VAL X  U/M X  COMP X | Y  6 variables; 64 events. 11 EPV. | Y | NI  5.0% excluded for incomplete record | Y  Variables with missing data exceeding 30% were excluded. For variables with less than 30% missing data, missing values were imputed using appropriate methods: the mean was used for continuous variables, and the mode was used for categorical variables. | Y  LASSO to identify variables | PN | Y | Y  5-fold cross-validation | Y | N/A |
| Li L *et al.* | **DEV √**  **INT VAL** X  EXT VAL X  U/M X  COMP X | N  9 variables; 63 events. 7 EPV. | Y | NI | Y  Variables with missing data exceeding 30% were excluded. For the remaining missing variables, imputation was performed using the MissForest package in R 4.3.3 to ensure the completeness and accuracy of the dataset. | Y  LASSO to identify variables | NI | Y | Y  5-fold cross-validation | NI | N/A |
| Li L *et al.* | **DEV √**  **INT VAL** X  EXT VAL X  U/M X  COMP X | Y  5 variables; 111 events. 22 EPV. | Y | NI  13.4% excluded for incomplete record | Y | Y  Boruta algorithm to identify variables | NI | Y | PN  Hold-out validation | NI | N/A |
| Li P *et al.* | **DEV √**  INT VAL X  EXT VAL X  U/M X  COMP X | N  9 variables; 54 events. 6 EPV. | Y | N  92/521 (17.7%) excluded for incomplete record | N | Y  LASSO regression analysis and Principal Component Analysis (PCA) to identify variables | NI | Y | N  No internal validation. | NI | N/A |
| Li X *et al.* | **DEV √**  **INT VAL** X  EXT VAL X  U/M X  COMP X | Y  10 variables; 201 events. 20 EPV. | Y | NI | NI | Y  RFE and 5-fold cross-validation to identify variables | NI | Y | Y  Bootstrap | NI | N/A |
| Lin Y *et al.* | **DEV √**  **INT VAL** X  EXT VAL X  U/M X  COMP X | Y  10 variables; 100 events. 10 EPV. | PN  The cut-off value was achieved using the ROC curve | NI  25/256 (9.8%) excluded for incomplete record | NI | N  univariate analysis to identify variables | NI | Y | Y  Bootstrap | Y | N/A |
| Liu X *et al.* | **DEV √**  **INT VAL** X  EXT VAL X  U/M X  COMP X | Y  8 variables; 131 events. 16 EPV. | Y | NI | NI | Y  LASSO, SVM-RFE and RF to identify variables | NI | Y | Y  Bootstrap and 10-fold cross-validation | NI | N/A |
| Lu X *et al.*  DEV | **DEV √**  **INT VAL √**  **EXT VAL √**  U/M X  COMP X | Y  12 variables; 182 events. 15 EPV. | Y | NI | NI | Y  ANOVA and LASSO to identify variables | NI | Y | PN  Hold-out validation | NI | N/A |
| Lu X *et al.*  EXT VAL |  | N for EXT VAL  91 sample size | Y |  |  |  | N/A  No model refitting |  |  |  |  |
| Luo H *et al.* | **DEV √**  **INT VAL** X  EXT VAL X  U/M X  COMP X | Y  11 variables; 160 events. 15 EPV. | Y | NI | NI | Y  elastic network, Lasso, Ridge, stepwise Cox, CoxBoost, RSF, SVM, partial least squares regression for Cox, GBM, and supervised principal components to identify variables | NI | Y | PN  Hold-out validation | NI | N/A |
| Ma Y *et al.* | **DEV √**  INT VAL X  EXT VAL X  U/M X  COMP X | Y  4 variables; 60 events. 15 EPV. | Y | NI | NI | N  LR to identify variables | NI | N  No calibration measures reported. | N  No internal validation | NI | N/A |
| Pang L *et al.* | **DEV √**  **INT VAL** X  EXT VAL X  U/M X  COMP X | N  5 variables; 44 events. 9 EPV. | Y | NI | NI | Y  LASSO, 10-fold cross-validation and multivariable LR to identify variables | NI | N  No calibration measures reported. | PN  Hold-out validation | NI | N/A |
| Pei W *et al.* | **DEV √**  **INT VAL** X  EXT VAL X  U/M X  COMP X | Y  7 variables; 426 events. 61 EPV. | Y | NI | Y  Multiple imputation was applied to handle missing data. | Y  Stepwise forward selection, LASSO and XGBoost to identify variables | NI | Y | PN  Hold-out validation | Y | N/A |
| Song X *et al.* | **DEV √**  **INT VAL** X  EXT VAL X  U/M X  COMP X | Y  5 variables; 104 events. 21 EPV. | Y | NI  69/757 (9.1%) excluded for incomplete record | Y  Multiple imputation was applied to variables with <20% missing data, whereas those with a proportion exceeding 20% were excluded. | Y  RF to identify variables | NI | Y | N  Hold-out validation; 2 EPV | Y | N/A |
| Sun Q *et al.* | **DEV √**  **INT VAL** X  EXT VAL X  U/M X  COMP X | NI  9 variables | Y | NI  102/1221 (8.4%) excluded for incomplete record | Y  Multiple imputation was applied to variables with <10% missing data, whereas those with a proportion exceeding 10% were excluded. | Y  LASSO regression analysis to identify variables | NI | Y | PN  Hold-out validation | Y | N/A |
| Wang M *et al.* | **DEV √**  **INT VAL** X  EXT VAL X  U/M X  COMP X | Y  11 variables; 243 events. 22 EPV. | Y | NI | Y  Variables missing more than 10% of the values were excluded; for variables missing less than 10% of the values, the data were interpolated (5 times interpolation). | N  Variables chosen on basis of univariate analysis. | NI | N  No calibration measures reported. | PN  Hold-out validation | Y | N/A |
| Wang Y *et al.* | **DEV √**  **INT VAL** X  EXT VAL X  U/M X  COMP X | N  6 variables; 26 events. 4 EPV. | Y | N  25/169 (14.8%) excluded for incomplete record | N  Excluded | Y  LASSO regression analysis to identify variables | NI | Y | Y  10-fold cross-validation | Y | N/A |
| Wei Z *et al.*  DEV | **DEV √**  **INT VAL √**  **EXT VAL √**  U/M X  COMP X | N  9 variables; 84 events. 9 EPV. | Y | NI | Y  The data were preprocessed by removing features with more than 20% missing values, and the remaining missing values were added to the dataset using the predictive mean matching method (PMM) for multiple imputations. | N  Variables chosen on basis of univariate analysis. | NI | N  No calibration measures reported. | Y  10-fold cross-validation | NI | N/A |
| Wei Z *et al.*  EXT VAL |  | N for EXT VAL  179 sample size | Y |  |  |  | N/A  No model refitting |  |  |  |  |
| Wei Z *et al.* | **DEV √**  **INT VAL** X  EXT VAL X  U/M X  COMP X | N  11 variables; 96 events. 9 EPV. | Y | NI | Y  The data were preprocessed by removing features with more than 10% missing values, and the remaining missing values were added to the dataset using the predictive mean matching method (PMM) for multiple imputations. | N  Univariate and multivariate Logistic Regression (LR) | NI | Y | Y  10-fold cross-validation | NI | N/A |
| Wen S *et al.*  DEV | **DEV √**  **INT VAL √**  **EXT VAL √**  U/M X  COMP X | PY  4 variables; 107 events. 27 EPV. | Y | NI | Y  For continuous variables, missing values were imputed using the mean if the data were normally distributed or the median if the data were skewed. For categorical variables, missing values were replaced with the most frequent category. If the proportion of missing values for a variable exceeded 30% of the total observations, the variable was excluded. | Y  LASSO regression analysis to identify variables | NI | Y | Y  10-fold cross-validation | Y | NI |
| Wen S *et al.*  EXT VAL |  | N for EXT VAL  173 sample size | Y |  |  |  | NI |  |  |  |  |
| Wu Z *et al.* | **DEV √**  **INT VAL** X  EXT VAL X  U/M X  COMP X | PY  5 variables; 55 events. 11 EPV. | Y | NI | NI | NI | NI | Y | PN  Hold-out validation | NI | N/A |
| Xie L *et al.* | **DEV √**  **INT VAL** X  EXT VAL X  U/M X  COMP X | PY  9 variables; 167 events. 19 EPV. | Y | NI | NI | Y  LASSO regression analysis to identify variables | NI | Y | Y  10-fold cross-validation | NI | N/A |
| Xie L *et al.* | **DEV √**  **INT VAL** X  EXT VAL X  U/M X  COMP X | PY  7 variables; 87 events. 12 EPV. | Y | NI | NI | Y  LASSO regression analysis to identify variables | NI | Y | Y  10-fold cross-validation | NI | N/A |
| Zhang J *et al.* | **DEV √**  **INT VAL** X  EXT VAL X  U/M X  COMP X | PY  8 variables; 106 events. 13 EPV. | Y | NI | NI | Y  LASSO and 10-fold cross-validation to identify variables | NI | Y | Y  5-fold cross-validation | NI | N/A |
| Zhang S *et al.* | **DEV √**  **INT VAL** X  EXT VAL X  U/M X  COMP X | N  10 variables; 37 events. 4 EPV. | Y | NI | Y  ELM was employed to replace the missing data. | Y  Pearson correlation coefficients and XGBoost to identify variables | NI | N  No calibration measures reported. | N  Hold-out validation; 1 EPV | NI | N/A |
| Zhang X *et al.*  DEV | **DEV √**  **INT VAL √**  **EXT VAL √**  U/M X  COMP X | PY  14 variables; 145 events. 10 EPV. | Y | NI | Y  Multiple imputation was applied to variables with ≤25% missing data, whereas those with a proportion exceeding 25% were excluded. | Y  LASSO regression analysis to identify variables | NI | N  No calibration measures reported. | N  Hold-out validation; 3 EPV | Y | N/A |
| Zhang X *et al.*  EXT VAL |  | N for EXT VAL  313 sample size | Y |  |  |  | N/A  No model refitting |  |  |  |  |
| Zhao H *et al.* | **DEV √**  **INT VAL** X  EXT VAL X  U/M X  COMP X | Y  4 variables; 86 events. 22 EPV. | Y | NI | NI | Y  univariate and multivariate stepwise logistic regression analysis, the 10-fold CV LASSO to identify variables | NI | N  No calibration measures reported. | N  Hold-out validation; 22 EPV | NI | N/A |

COMP=study which compares two or more models; DEV=model development study; EPV=events per variable; EXT VAL =study with external validation of a model; INT VAL= study with internal model validation; N=no; NEI=not enough information; NI=no information; PN=probably no; PY=probably yes; U/M =study which updates or modifies a model; Y=yes.

**Risk of bias assessment –key (informed by PROBAST)**

| *Appropriate data source?* | Y if prospective cohort with consecutive aortic dissection (AD) patients (or all within a specified timeframe).  PY if retrospective analysis with consecutive patients or prospective cohort (limited details).  NI if single-center study with insufficient information on patient selection.  PN if subgroup analysis from a larger AD or acute aortic syndrome cohort. |
| --- | --- |
| *Were all inclusions and exclusion appropriate?* | PY if inclusion criteria were clearly defined and appropriate for review question (e.g., studies on risk prediction models for in-hospital mortality, malperfusion, or complications in AD).  NI if eligibility criteria were inconsistently reported (e.g., mixed AD types (Stanford A/B), unclear diagnostic criteria, or mixed model development/validation purposes).  PN if exclusion criteria were poorly documented (e.g., prior aortic surgery, traumatic AD, iatrogenic dissection). |
| *Applicability* –do participants/setting match the review question? | Not included in table. Most studies focused on AD risk prediction, but some models were derived from mixed cardiovascular emergency populations and later applied specifically to AD. These are flagged for "indirectness" (GRADE) in the discussion. |
| *Predictors defined and assessed in similar way for all participants?* | Y if reference made to standard criteria used in all patients (e.g., imaging protocols, laboratory standards).  PY if single centre. For some criteria there is a standard way of measuring (e.g. aortic diameter on CT, biomarkers), others are not prone to measurement issues (e.g. age, sex, history of hypertension). Some information is unlikely to be reported, e.g., how co-morbidities were defined.  PY if multicentre but with a standardised data collection protocol.  NI if not able to tell if single centre and no other information.  N if specific statement that variables were measured in different ways across sites or timepoints. |
| *Predictor assessment made without knowledge of outcome data?* | Y if clear that all predictors measured at presentation or pre-operatively, before the outcome occurred (e.g., admission demographics, imaging, labs).  PY if appears that (at least some) predictors were measured at admission. Less important for fixed predictors (age, sex, medical history).  NI if no details on timing of predictor assessment relative to outcome. |
| *All predictors available at the time the model is intended to be used?* | Y if all predictors available at initial presentation or time of decision-making (e.g., clinical variables, imaging findings from initial scan).  N if included events or data occurring after initial management decisions (e.g., procedural complications, response to initial therapy) unknown at baseline. |
| *Was the outcome determined appropriately?* | Y if outcome confirmed by definitive methods (e.g., surgery, autopsy, imaging review by adjudication committee).  PY if based on clinical diagnosis documented in medical records (e.g., in-hospital mortality, malperfusion syndrome) without strict adjudication protocol.  NI if outcome defined solely by diagnostic codes or retrospective record review without active confirmation. |
| *Standard outcome definition?* | Y if outcome (e.g., mortality, composite complication) was clearly defined and assessed within a specified timeframe relevant to AD (e.g., in-hospital, 30-day, 1-year).  PY if outcome defined but timeframe unspecified or unclear.  NI if no clear definition provided for the outcome. |
| *Were predictors excluded from the outcome definition?* | This is always Y. Predictors do not form part of outcome assessment. |
| *Was the outcome defined and determined in a similar way for all participants?* | Y if explicit statement of a standardized outcome assessment protocol across all patients.  PY if a standard definition is given and/or the study is single-center, suggesting consistent practice. |
| *Was the outcome determined without knowledge of predictor information?* | NI if no information on blinding of outcome assessors.  N if outcome assessors were aware of predictor status (e.g., known high-risk imaging features or biomarker levels). |
| *Appropriate time interval between predictor and outcome assessment?* | Y if follow-up period was sufficient to capture the outcome of interest (e.g., entire hospitalization for in-hospital mortality, ≥30 days for short-term survival).  PY if likely adequate (e.g., median follow-up reported and seems sufficient).  NI no (or unclear) information given on length of follow-up.  PN if follow-up was too short to reasonably assess the outcome (e.g., assessing 30-day mortality with only 24-hour follow-up).  This was somewhat subjective as sometimes only a mean or median (with or without a range was stated) and the minimum follow-up period for all patients was not known. |
| *Was there a reasonable number of participants with the outcome?* | Development studies  Y if >20 events per variable for candidate predictors  PY if > 10 events per variable  NI no details or unclear how many candidate variables  PN if number of candidate variables unclear but small sample size  N <10 events per variable  Validation studies  Y at least 100 participants with outcome.  PY if events not stated but very large sample size (e.g. >1000)  NI number of events not stated  N less than 100 participants with outcome |
| *Were continuous and categorical predictors handled appropriately?* | Development studies  Y if continuous predictors were used as continuous in the model, or if dichotomization/categorization was based on pre-defined, clinically accepted cut-offs.PN - appears that one or more cut-offs based on study data  NI no details provided on handling of continuous variables.  PN appears that one or more cut-offs were determined based on the study data (e.g., using median/optimal cut-point analysis).  N if dichotomization of continuous predictors was based on study data without adjustment for optimism (e.g., via internal validation and shrinkage).  Validation studies  Y if the model was applied exactly as developed, using the same variable definitions and cut-offs.  PY appears that the same model is being used but not explicitly stated.  PN appears that some model variables or their definitions have been changed for the validation.  N some model variables or cut-offs were clearly changed from the original development. |
| *Were all enrolled participants included in the analysis?* | Y explicit statement that all eligible patients were included in the analysis. If some were excluded, evidence provided that their characteristics were similar to those included.N-clear statement that >10% patients lost to follow-up (or with missing predictor information) were excluded from the analysis and/or that patient characteristics differed between in-and excluded  NI no details, or a small proportion (<10%) of patients excluded but no information on similarity between included and excluded groups.  N clear statement that >10% of patients (due to loss to follow-up or missing data) were excluded from analysis, and/or that characteristics differed significantly between included and excluded groups. |
| *Were participants with missing data handled appropriately?* | 1. an explicit statement that all enrolled patients were included in the final analysis or studies reported an appropriate method for handling missing data, e.g. by using multiple imputation.   NI- no details on handling of missing data  N- patients lost to follow-up simply excluded (and no details on similarity of patients characteristics between in- and excluded)  N/A in studies where all patients had been included in analysis (or if excluded had similar characteristics to included). |
| *Was selection of predictors based on univariate analysis avoided? (DEV only)* | Y- if predictors identified through multivariate analysis  NI –no details on how predictors were selected  N- if predictors selected on the basis of univariable analysis before multivariable modelling |
| *Were complexities in the data (e.g. censoring, competing risks, sampling of control participants) accounted for appropriately?* | Development studies  Y- time to event analysis used (e.g. Cox analysis)  NI-no details on type of analysis  PN- logistic regression model used/insufficient information  N-no time-to event analysis used  Validation studies  N/A as none of the validation studies undertook calibration and model refitting. |
| *Were relevant model performance measures evaluated appropriately?* | Y-both a discrimination and calibration statistic reported  N-only one of the above or none presented |
| *Were model overfitting and optimism in model performance accounted for? (DEV only)* | Y-a form of internal validation included (e.g. bootstrapping or cross-validation); where included should adjust or shrink the model predictive performance estimates and predictor effects in the final model  PN- a split sample approach used with >20 events per candidate variable  N-no form of internal validation or a split sample approach with <20 events per candidate variable |
| *Do predictors and their assigned weights in the final model correspond to the results from the reported multivariable analysis? (DEV only)* | Y-regression co-efficients used to estimate contribution of each variable to the risk  PY- score scale based on regression co-efficients (with no further details)  NI-no information on how weights assigned  N-inappropriate method, e.g. assigning points based on relative risk or degree of separation of Kaplan-Meier curves |

DEV=model development study; N=no; NEI=not enough information; NI=no information; PN=probably no; PY=probably yes; Y=yes.
