# Supplementary material for: Machine learning approaches for risk prediction in aortic dissection: a systematic review and meta-analysis
Source: Front Cardiovasc Med. 2026 Mar 26;13:1777734. doi: 10.3389/fcvm.2026.1777734 (PMC13062221; doi:10.3389/fcvm.2026.1777734)
Supplement: Supplementary file 12 [file Table11.docx]

**Supplementary Table S11. Risk of bias appraisal results of eligible articles adapted from PROBAST**

| **Study** | **ROB** | | | | **Applicability** | | | **Overall** | |
| --- | --- | --- | --- | --- | --- | --- | --- | --- | --- |
|  | **Participants** | **Predictors** | **Outcome** | **Analysis** | **Participants** | **Predictors** | **Outcome** | **ROB** | **Applicability** |
| Cai H 2025 | ＋ | ＋ | + | － | + | + | + | － | + |
| Chen H 2023 | ＋ | ＋ | ? | － | + | + | + | － | + |
| Chen Q 2021 | ＋ | ＋ | ＋ | － | + | + | + | － | + |
| Chen Q 2025 | ＋ | ＋ | ＋ | － | + | + | + | － | + |
| Chen Z 2025 | ＋ | ＋ | ? | ? | + | + | + | ? | + |
| Dai A 2023 | ＋ | ＋ | ? | ? | + | + | + | ? | + |
| Dong Y 2021 | ＋ | ＋ | ? | － | + | + | + | － | + |
| Guo T 2021 | ＋ | ＋ | ＋ | － | + | + | + | － | + |
| Guo Z 2022 | ＋ | ＋ | ＋ | ? | + | + | + | ? | + |
| He X 2025 | ＋ | ＋ | ＋ | － | + | + | + | － | + |
| Jiang Y 2023 | ＋ | ＋ | ＋ | － | + | + | + | － | + |
| Jin Z 2025 | ＋ | ＋ | ? | ? | + | + | + | ? | + |
| Jin Z 2025 | ＋ | ＋ | ? | ? | + | + | + | ? | + |
| Lei J 2024 | ＋ | ＋ | ＋ | － | + | + | + | － | + |
| Li K 2025 | ＋ | ＋ | ? | － | + | + | + | － | + |
| Li L 2025 | ＋ | ＋ | ? | － | + | + | + | － | + |
| Li L 2025 | ＋ | ＋ | ? | － | + | + | + | － | + |
| Li P 2024 | ＋ | ＋ | ＋ | － | + | + | + | － | + |
| Li X 2022 | ＋ | ＋ | ? | ? | + | + | + | ? | + |
| Lin Y 2023 | ＋ | ＋ | ＋ | － | + | + | + | － | + |
| Liu X 2024 | ＋ | ＋ | ? | ? | + | + | + | ? | + |
| Lu X 2024 | ＋ | ＋ | ? | － | + | + | + | － | + |
| Luo H 2025 | ＋ | ＋ | ? | － | + | + | + | － | + |
| Ma Y 2024 | ＋ | ＋ | ? | － | + | + | + | － | + |
| Pang L 2024 | ＋ | ＋ | ＋ | － | + | + | + | － | + |
| Pei W 2021 | ＋ | ＋ | ＋ | － | + | + | + | － | + |
| Song X 2024 | ＋ | ＋ | ＋ | － | + | + | + | － | + |
| Sun Q 2024 | ＋ | ＋ | ＋ | － | + | + | + | － | + |
| Wang M 2023 | ＋ | ＋ | ? | － | + | + | + | － | + |
| Wang Y 2023 | ＋ | ＋ | ＋ | － | + | + | + | － | + |
| Wei Z 2025 | ＋ | ＋ | ? | － | + | + | + | － | + |
| Wei Z 2025 | ＋ | ＋ | ? | － | + | + | + | － | + |
| Wen S 2025 | ＋ | ＋ | ? | ? | + | + | + | ? | + |
| Wu Z 2023 | ＋ | ＋ | ＋ | － | + | + | + | － | + |
| Xie L 2024 | ＋ | ＋ | ＋ | ? | + | + | + | ? | + |
| Xie L 2024 | ＋ | ＋ | ? | ? | + | + | + | ? | + |
| Zhang J 2024 | ＋ | ＋ | ＋ | ? | + | + | + | ? | + |
| Zhang S 2025 | ＋ | ＋ | ＋ | － | + | + | + | － | + |
| Zhang X 2025 | ＋ | ＋ | ＋ | － | + | + | + | － | + |
| Zhao H 2021 | ＋ | ＋ | ? | － | + | + | + | － | + |

“+” indicates low ROB/low concern regarding applicability; “－” indicates high ROB/low concern regarding applicability; and “?” indicates unclear ROB/low concern regarding applicability

ROB risk of bias
