# Supplementary figures and images for: Machine learning approaches for risk prediction in aortic dissection: a systematic review and meta-analysis
Source: Front Cardiovasc Med. 2026 Mar 26;13:1777734. doi: 10.3389/fcvm.2026.1777734 (PMC13062221; doi:10.3389/fcvm.2026.1777734)

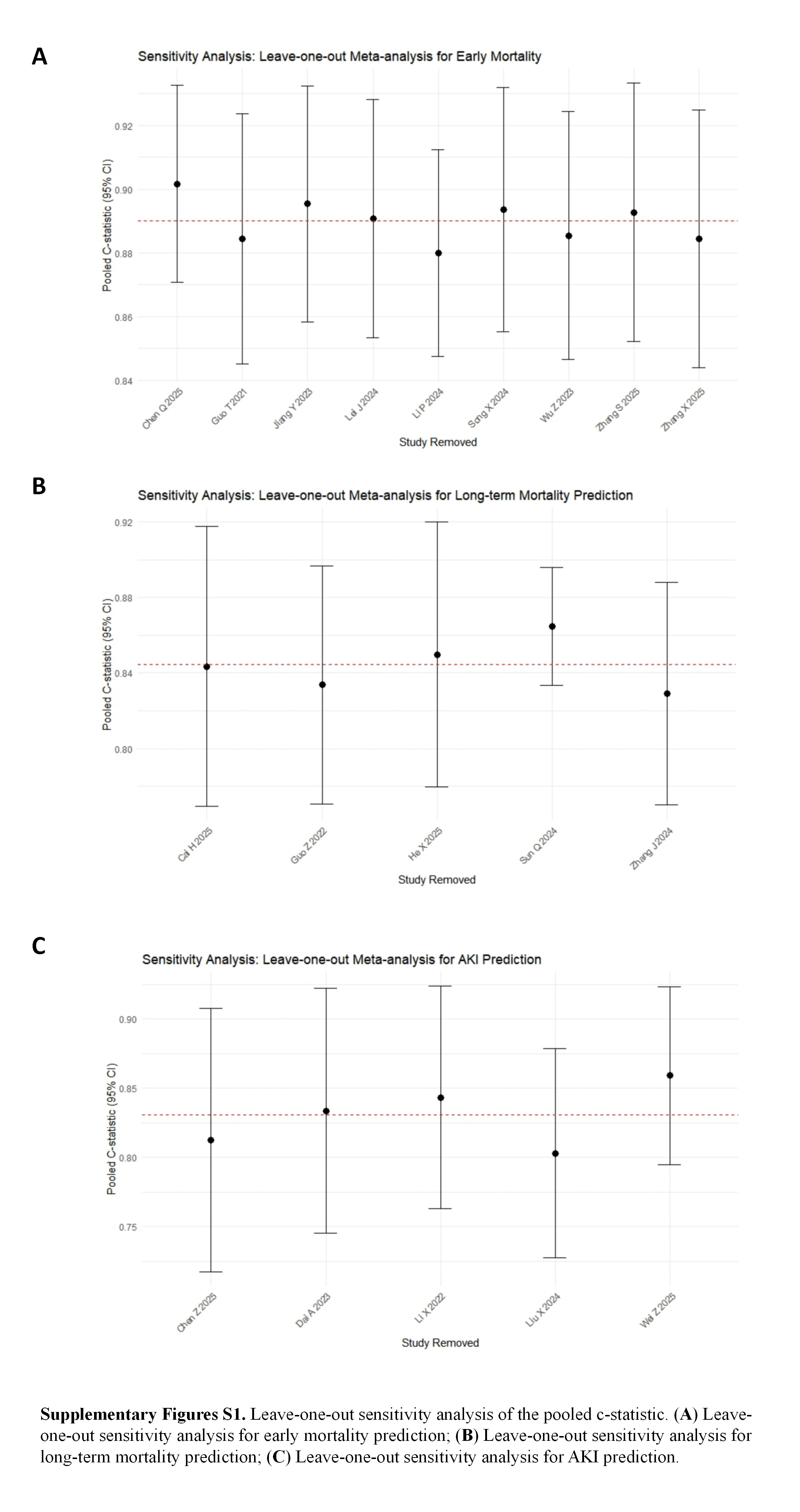

Supplement: Supplementary file 13 [file Image1.tif]

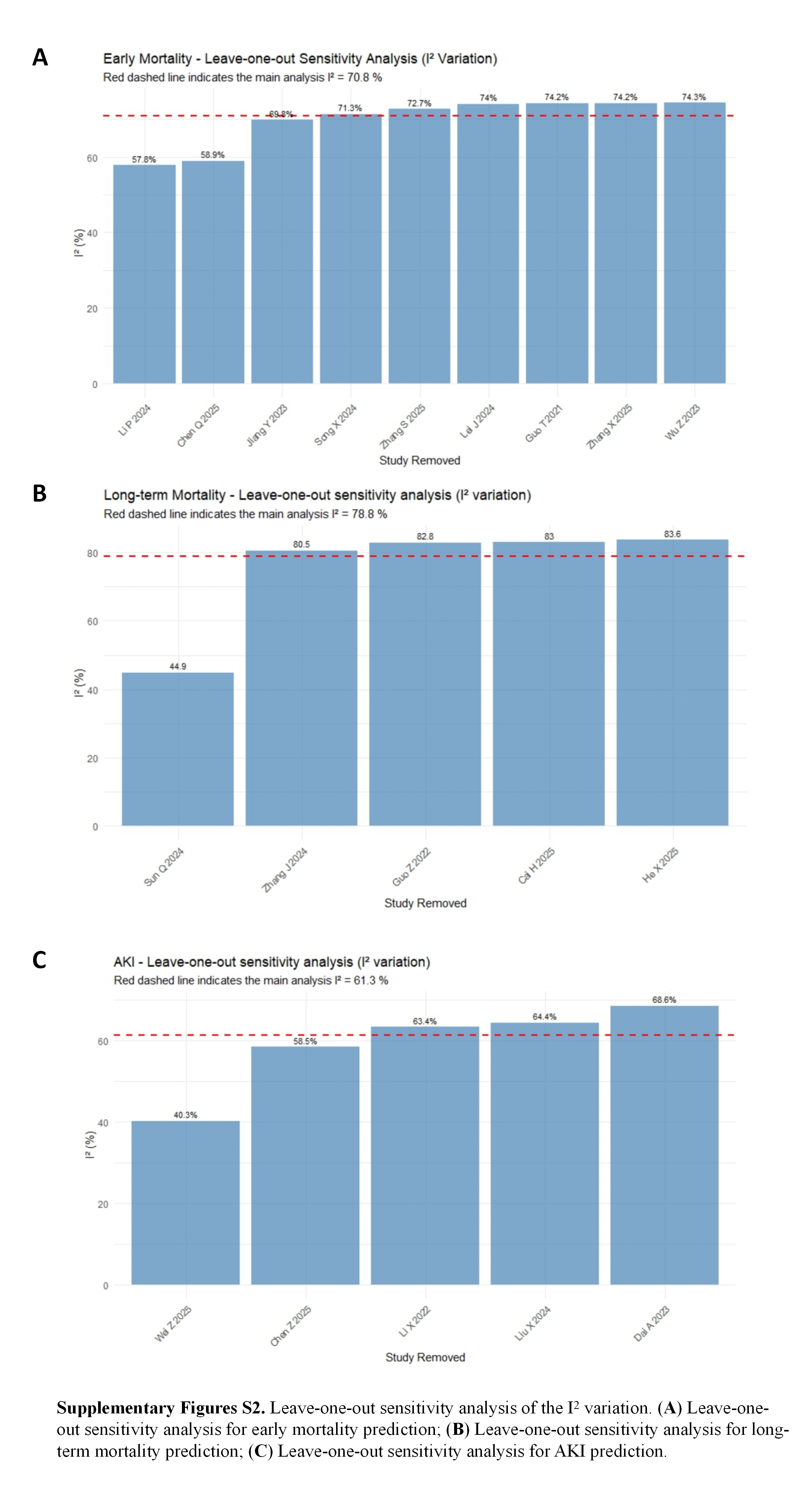

Supplement: Supplementary file 14 [file Image2.tif]

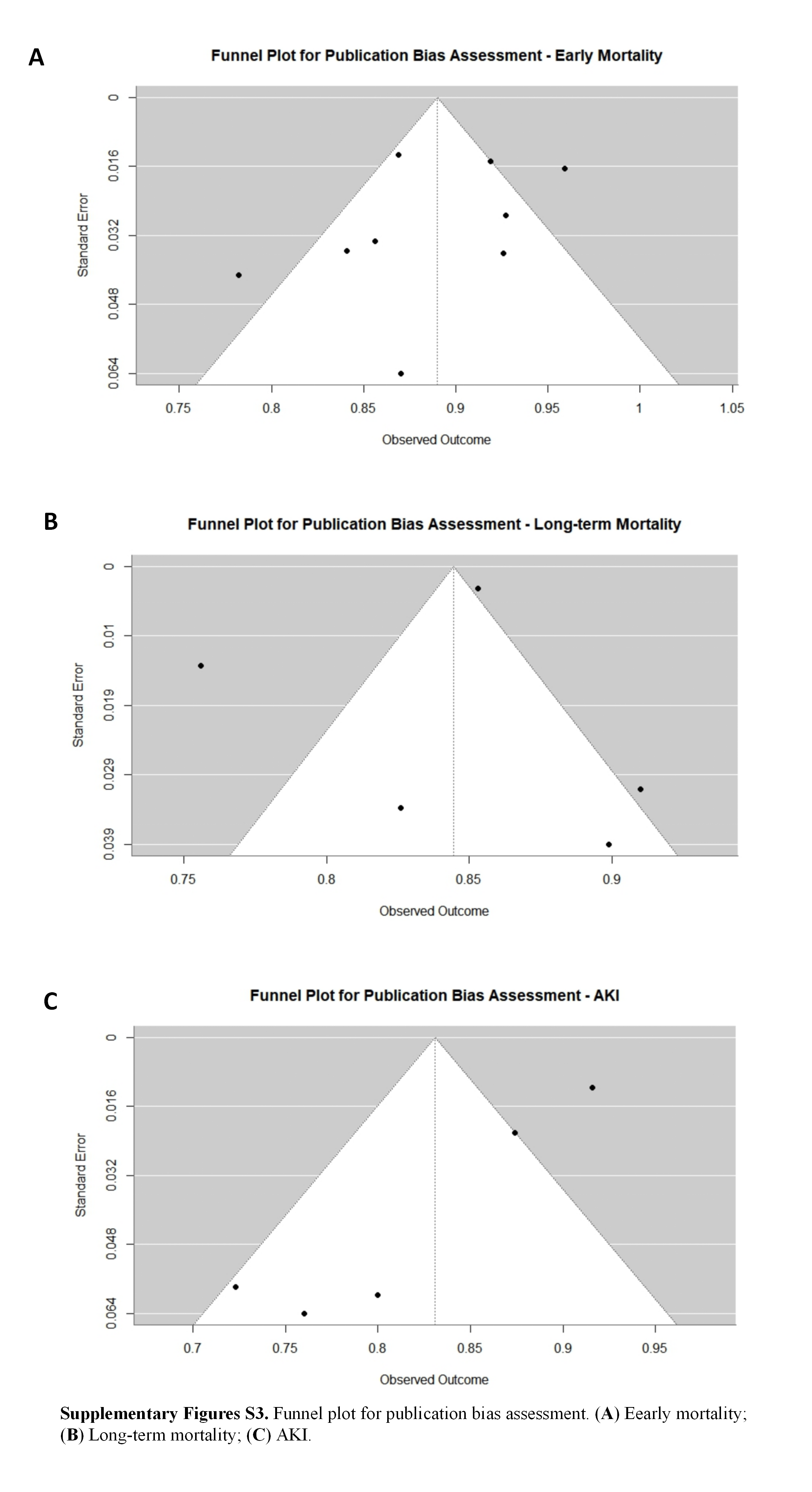

Supplement: Supplementary file 15 [file Image3.tif]
